# Subtype‐specific epidemiology of lymphoid malignancies in Taiwan compared to Japan and the United States, 2002‐2012

**DOI:** 10.1002/cam4.1762

**Published:** 2018-10-09

**Authors:** Bor‐Sheng Ko, Li‐Ju Chen, Huai‐Hsuan Huang, Yao‐Chun Wen, Chi‐Yin Liao, Ho‐Min Chen, Fei‐Yuan Hsiao

**Affiliations:** ^1^ Division of Hematology Department of Internal Medicine National Taiwan University Hospital Taipei Taiwan; ^2^ Health Data Research Center National Taiwan University Taipei Taiwan; ^3^ Graduate Institute of Clinical Pharmacy National Taiwan University Taipei Taiwan; ^4^ School of Pharmacy National Taiwan University Taipei Taiwan; ^5^ Department of Pharmacy National Taiwan University Hospital Taipei Taiwan

**Keywords:** age‐standardized rate, epidemiology, lymphoma, Taiwan Cancer Registry Database

## Abstract

**Background:**

There are many unrevealed parts regarding lymphoma etiology. Previous studies suggested differences in lymphoma epidemiology among countries existed; however, some were one‐center studies that were not enough to represent the whole population.

**Objective:**

To provide epidemiological information on lymphoma within Taiwanese and to compare the data with that in Japan and the United States.

**Methods:**

We used Taiwan Cancer Registry Database as our data source. Patients with lymphoma were identified through the ICD‐O‐3 codes and those with non‐Hodgkin lymphoma (NHL) were categorized into three major types and 13 subtypes according to 2008 WHO classification. Incidence of lymphoma was adjusted according to the 2000 world standard population.

**Results:**

During 2002‐2012, 21 929 cases were diagnosed with four major types of lymphoma in Taiwan. Aggressive B‐cell lymphoma (52.21%, N = 11 450) was the most common type of NHL. Median age at diagnosis of aggressive B‐cell lymphoma was the eldest (63.0‐65.0 years). Male excess in T/NK‐cell lymphoma was the most obvious (sex ratio: 1.39‐2.07). The incidence of NK/T‐cell lymphoma, nasal type, was higher (male: 0.16‐0.34 per 100 000, female: 0.06‐0.16 per 100 000) in Taiwan than that in the United States and Japan.

**Conclusion:**

This is the first population‐based study in Taiwan to investigate subtype‐specific epidemiology of lymphoma. The incidence rates of lymphoma in Taiwan are mostly lower than those in the United States and higher or comparable to those in Japan except for NK/T‐cell lymphoma, nasal type, whose age‐adjusted incidence in Taiwan is the highest.

## 1 INTRODUCTION

Large‐scale and population‐based disease epidemiology is critical to inform resource allocation and research prioritization. However, current evidence is scarce, particularly for lymphoid malignancies. Lymphoid malignancies encompass a group of cancers varying enormously from the morphology, histology, immuno‐phenotypes, genotypes, as well as the clinical features, treatment, and outcomes.^1^, ^2^

Recently, more attention has been paid to evaluate the distribution of lymphoma subtypes and to compare their incidences and prevalences between East and West countries, which might help to shed some light on the intricate etiology and associated genetic or environmental risk factors of lymphoid malignancies. As stated in a previous research, the incidence rates of mature T/natural killer (NK)‐cell lymphoma and extranodal marginal zone B‐cell lymphoma of mucosa‐associated lymphoid tissue type (MALT) were higher in Asian populations than those in North America and Europe.^2^, ^3^ In contrast, the incidences of follicular lymphoma and chronic lymphocytic leukemia (CLL) were relatively lower in Asians.^2^, ^3^ The epidemiology of Epstein‐Barr virus (EBV) and human T‐lymphoblastic virus‐1 (HTLV‐1) around the world as well as several genetic factors might be the explanation of discrepancy in incidence between Asia and West.^4^, ^5^

Although several studies have assessed the subtype‐specific incidence rates of lymphoid malignancies between Western and Asian countries,^6^, ^7^, ^8^, ^9^ many of them were limited to one‐center studies and did not represent the whole population. In addition, because Taiwan is a multiethnic society with Austronesian heritage, the genetic composition of a Taiwanese population might be different from that of a Japanese or a South Korean population to some extent. Therefore, our study aims to conduct an epidemiological study on lymphoid malignancies within the Taiwanese population using Taiwan Cancer Registry Database (TCRD) and to compare the data acquired from Taiwan with that from Japan and the United States^7^ in order to reveal complicated etiology of lymphoma.

## 2 MATERIALS AND METHODS

### 2.1 Data source

We collected information of the newly diagnosed lymphoma cases between 2002 and 2012 from the TCRD. The TCRD is a population‐based cancer registry established and funded by the Ministry of Health and Welfare (MOHW) in 1979. A Cancer Registry Advisory Board was organized and responsible for standardizing the procedures, definitions of terminology, and coding of the reporting system for the registry. The TCRD covers cancer patients with all ages who have been admitted to or have gone to hospitals with more than 50 beds. With 98.4% completeness and 91.5% diagnoses confirmed with histological and/or cytological verification, the TCRD has been used as a tremendous tool to enhance the quality of cancer care.^10^, ^11^ All cancer types were coded in the TCRD based on the International Classification of Diseases for Oncology, the Third Edition (ICD‐O‐3)^12^ since 2002.

### 2.2 Ethical statement

The protocol of our study was approved by the Research Ethics Committee of National Taiwan University Hospital (registration number, 201604051W).

### 2.3 Study population

Patients with Hodgkin lymphoma (HL) and non‐Hodgkin lymphoma (NHL) were identified using the ICD‐O‐3 codes reported by registries. Those with NHL were further categorized into three major types (aggressive B‐cell lymphoid neoplasm, indolent B‐cell lymphoid neoplasms, and T/NK‐cell lymphoid neoplasm) and 13 frequently seen subtypes according to the 2008 WHO classification system,^1^, ^2^ including diffuse large B‐cell lymphoma (DLBCL), follicular lymphoma (FL), chronic lymphocytic leukemia/small lymphocytic lymphoma (CLL/SLL), Burkitt lymphoma (BL), mantle cell lymphoma (MCL), marginal zone B‐cell lymphoma (MZBCL, including MALT), peripheral T‐cell lymphoma‐NOS (PTCL‐NOS), mycosis fungoides (MF), cutaneous T‐cell lymphoma (CTCL), anaplastic large T/null‐cell lymphoma (ALCL), angioimmunoblastic T‐cell lymphoma (AITL), NK/T‐cell lymphoma, nasal type (NK/TCL), and adult T‐cell leukemia/lymphoma (ATLL). The disease codes used in this study are presented in Table 1. Patients without pathologically confirmed or aged younger than 20 years at the diagnosis were excluded.

**Table 1:** Disease coding of Hodgkin and non‐Hodgkin lymphoma

Disease	ICD‐O‐3 code
Hodgkin lymphoma	96503‐96673
Non‐Hodgkin lymphoma	
Aggressive B‐cell lymphoid neoplasm	96733/96793/96803/96843/96873/96883/97123/97353/97373/97383/98263
Diffuse large B‐cell lymphoma (DLBCL)	96803/96843/96883/97123/97353/97373/97383
Burkitt lymphoma (BL)	96873/98263
Mantle cell lymphoma (MCL)	96733
Indolent B‐cell lymphoid neoplasm	95973/96713/96753/96893/96903/96913/96953/96983/96993/97613/98233
Follicular lymphoma (FL)	95973/96753/96903/96913/96953/96983
Chronic lymphocytic leukemia/small lymphocytic lymphoma (CLL/SLL)	98233
Marginal zone B‐cell lymphoma (MZBCL)	96893/96993
T/NK‐cell lymphoid neoplasm	97003‐97023/97053/97083/97093/97143/97163‐97193/97263/97293/98273/98313/98343/98373/99483
Peripheral T‐cell lymphoma‐NOS (PTCL‐NOS)	97023
Mycosis fungoides (MF)	97003
Cutaneous T‐cell lymphoma (CTCL)	97013/97093/97183/97263
Anaplastic large T/null‐cell lymphoma (ALCL)	97143
Angioimmunoblastic T‐cell lymphoma (AITL)	97053
NK/T‐cell lymphoma, nasal type (NK/TCL)	97193
Adult T‐cell leukemia/lymphoma (ATLL)	98273

NOS, not otherwise specified.

### 2.4 Statistical analysis

Number of incidence, median age of diagnosis, and sex ratio were calculated and reported annually for patients with HL as well as three major types and 13 subtypes of NHL during the 11 years of study period (from 2002 to 2012). Crude rates and age‐standardized rates (ASRs) adjusted by the 2000 world standard population as defined by the World Health Organization^13^ were both presented as cases per 100 000 persons. We further investigated the distribution (proportion) of both sexes among all subtypes and compared the age‐standardized rates with those reported in Japan and the United States. All data in this study were analyzed using SAS^®^ software, version 9.4 (SAS Institute, Cary, NC, USA).

## 3 RESULTS

### 3.1 Overall incidence

**Figure 1:**
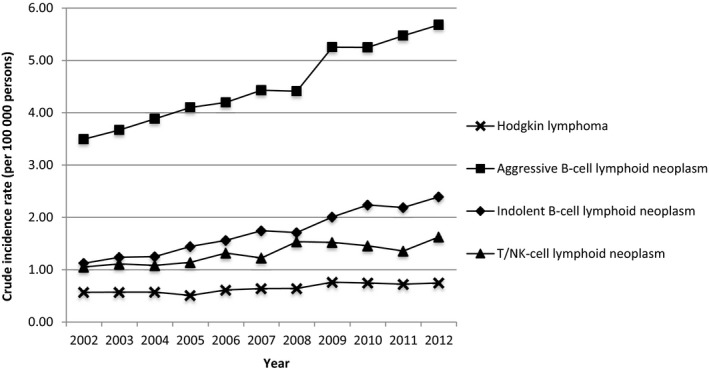
Crude incidence rates of four major types of lymphoma in Taiwan between the years 2002‐2012

**Figure 2:**
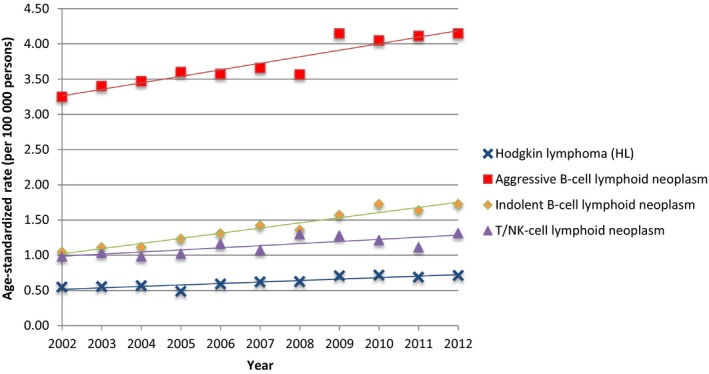
Age‐standardized rates (ASRs) and trends of four major types of lymphoma in Taiwan between 2002 and 2012

From 2002 to 2012, a total of 21 929 cases were diagnosed with four major types of lymphoma in Taiwan. The crude incidence rates and age‐standardized rates of four major types and most of the subtypes of lymphomas increased during the 11‐year study period (Figures 1, 2 and S1, S2). Among the four major types, aggressive B‐cell lymphoma (52.21%, N = 11 450) was consistently the most common lymphoma followed by indolent B‐cell lymphoma (25.24%, N = 5535) and T/NK‐cell lymphoid neoplasm (15.12%, N = 3315). It was noteworthy that the crude incidence rate of indolent B‐cell lymphoma had a more than twofold increase in 11 years (from 1.12 per 100 000 persons in 2002 to 2.39 per 100 000 persons in 2012). The detailed percentages of four major types of lymphoma are demonstrated annually in Figure S3. Hodgkin lymphoma remained the least occurred lymphoma (7.43%, N = 1629) throughout the study period although we observed a slight increase in crude incidence rates from 0.57 in 2002 to 0.75 in 2012 (Figure 1).

As for the 13 subtypes of NHL, DLBCL accounted for almost half of the lymphoma cases (44.18%, N = 9688) and occurred far more frequently than the following four frequently diagnosed subtypes, including FL (9.47%, N = 2076), MZBCL (9.14%, N = 2004), CLL/SLL (5.66%, N = 1241), and PTCL‐NOS (4.73%, N = 1038). The lowest incidence was seen in ATLL (0.17%, N = 38), and some of the figures regarding this category were unavailable due to insufficient number of patients diagnosed in the calendar year (Table 2).

**Table 2:** Number of incident cases and age‐standardized rates (ASRs) of patients with HL, three major types and 13 subtypes of NHL in Taiwan between 2002 and 2012

Disease	Number of incidence/ASR	2002			2003			2004			2005			2006			2007			2008			2009			2010			2011			2012		
		M	F	Total	M	F	Total	M	F	Total	M	F	Total	M	F	Total	M	F	Total	M	F	Total	M	F	Total	M	F	Total	M	F	Total	M	F	Total
HL	Number of Incidence	81	47	128	76	53	129	84	46	130	69	47	116	88	52	140	84	63	147	86	62	148	92	84	176	104	69	173	105	63	168	101	73	174
	ASR	0.68	0.42	0.55	0.66	0.45	0.56	0.72	0.41	0.57	0.57	0.40	0.49	0.74	0.44	0.59	0.69	0.56	0.62	0.71	0.54	0.63	0.72	0.71	0.71	0.86	0.57	0.72	0.83	0.56	0.69	0.80	0.64	0.71
NHL																																		
Aggressive B‐cell lymphoid neoplasm	Number of Incidence	427	360	787	471	359	830	496	385	881	547	387	934	539	421	960	575	442	1017	592	424	1016	659	555	1214	692	524	1216	695	576	1271	729	595	1324
	ASR	3.46	3.04	3.25	3.80	2.98	3.40	3.85	3.08	3.47	4.22	2.99	3.60	3.97	3.16	3.57	4.16	3.15	3.66	4.20	2.95	3.57	4.60	3.80	4.15	4.70	3.41	4.05	4.59	3.66	4.11	4.76	3.56	4.15
DLBCL	Number of Incidence	364	305	669	395	322	717	407	326	733	443	338	781	450	364	814	470	393	863	492	373	865	548	479	1027	574	458	1032	564	507	1071	600	516	1116
	ASR	2.95	2.58	2.76	3.14	2.66	2.91	3.11	2.58	2.85	3.37	2.59	2.98	3.26	2.71	3.00	3.35	2.80	3.07	3.44	2.57	2.99	3.81	3.15	3.48	3.79	2.98	3.38	3.68	3.18	3.42	3.84	3.02	3.42
BL	Number of Incidence	20	22	42	26	13	39	30	19	49	32	18	50	33	25	58	41	12	53	31	17	48	30	21	51	35	14	49	24	15	39	38	14	52
	ASR	0.17	0.20	0.18	0.23	0.12	0.18	0.28	0.18	0.23	0.29	0.16	0.22	0.29	0.22	0.26	0.34	0.09	0.21	0.28	0.15	0.21	0.25	0.16	0.21	0.33	0.09	0.21	0.22	0.11	0.16	0.31	0.12	0.21
MCL	Number of Incidence	18	5	23	19	3	22	22	6	28	30	6	36	26	6	32	29	13	42	29	7	36	42	14	56	42	14	56	43	13	56	31	9	40
	ASR	0.14	0.04	0.09	0.17	0.02	0.09	0.16	0.05	0.11	0.24	0.05	0.14	0.19	0.04	0.12	0.22	0.09	0.15	0.20	0.05	0.12	0.28	0.09	0.18	0.27	0.09	0.18	0.27	0.08	0.17	0.19	0.05	0.12
Indolent B‐cell lymphoid neoplasm	Number of Incidence	139	114	253	155	125	280	139	145	284	195	134	329	188	169	357	211	190	401	199	195	394	248	216	464	255	263	518	265	243	508	304	254	558
	ASR	1.15	0.95	1.05	1.21	1.00	1.11	1.07	1.15	1.11	1.48	0.99	1.23	1.39	1.23	1.31	1.5	1.34	1.42	1.37	1.35	1.36	1.7	1.46	1.57	1.74	1.73	1.72	1.74	1.54	1.64	1.91	1.55	1.72
FL	Number of Incidence	68	50	118	90	68	158	80	64	144	104	70	174	102	81	183	100	93	193	94	83	177	116	93	209	108	123	231	124	116	240	136	113	249
	ASR	0.56	0.41	0.48	0.70	0.54	0.62	0.62	0.51	0.57	0.77	0.51	0.64	0.74	0.59	0.66	0.72	0.67	0.70	0.66	0.57	0.62	0.80	0.62	0.71	0.74	0.80	0.77	0.81	0.74	0.77	0.85	0.69	0.77
CLL/SLL	Number of Incidence	55	27	82	57	28	85	64	40	104	66	37	103	63	36	99	73	28	101	74	43	117	86	44	130	94	46	140	97	55	152	72	56	128
	ASR	0.45	0.23	0.34	0.45	0.22	0.34	0.49	0.31	0.40	0.52	0.28	0.40	0.46	0.26	0.36	0.51	0.19	0.35	0.50	0.30	0.40	0.58	0.29	0.43	0.63	0.29	0.45	0.64	0.33	0.47	0.44	0.33	0.39
MZBCL	Number of Incidence	67	63	130	58	53	111	52	79	131	79	60	139	70	83	153	101	95	196	90	108	198	112	117	229	118	131	249	109	112	221	132	115	247
	ASR	0.55	0.53	0.54	0.46	0.43	0.45	0.40	0.63	0.51	0.61	0.45	0.53	0.53	0.61	0.57	0.71	0.66	0.69	0.61	0.75	0.68	0.76	0.79	0.78	0.80	0.87	0.83	0.72	0.71	0.72	0.83	0.71	0.77
T/NK‐cell lymphoid neoplasm	Number of Incidence	140	97	237	171	80	251	151	95	246	177	83	260	201	101	302	181	100	281	230	124	354	230	122	352	220	118	338	197	118	315	225	154	379
	ASR	1.14	0.82	0.99	1.39	0.68	1.04	1.20	0.77	0.99	1.37	0.67	1.02	1.56	0.77	1.16	1.39	0.77	1.08	1.72	0.89	1.30	1.66	0.91	1.28	1.6	0.85	1.22	1.38	0.87	1.11	1.6	1.03	1.31
PTCL‐NOS	Number of Incidence	60	36	96	58	31	89	49	37	86	59	22	81	60	36	96	55	22	77	75	38	113	71	30	101	84	32	116	57	28	85	58	40	98
	ASR	0.49	0.31	0.40	0.46	0.26	0.36	0.38	0.29	0.34	0.45	0.18	0.32	0.46	0.27	0.37	0.40	0.16	0.28	0.54	0.27	0.40	0.51	0.20	0.35	0.59	0.21	0.40	0.40	0.17	0.28	0.38	0.25	0.31
MF	Number of Incidence	9	6	15	*	*	13	11	4	15	10	5	15	19	4	23	*	*	14	12	9	21	16	12	28	11	10	21	11	15	26	16	14	30
	ASR	0.07	0.05	0.06	*	*	0.05	0.08	0.03	0.06	0.07	0.04	0.06	0.15	0.03	0.09	*	*	0.06	0.09	0.07	0.08	0.12	0.09	0.10	0.08	0.07	0.07	0.07	0.11	0.09	0.12	0.10	0.11
CTCL	Number of Incidence	4	8	12	15	3	18	*	*	6	14	3	17	12	5	17	3	4	7	9	5	14	6	6	12	*	*	6	6	4	10	*	*	5
	ASR	0.03	0.06	0.05	0.13	0.02	0.08	*	*	0.03	0.10	0.02	0.06	0.09	0.04	0.07	0.02	0.03	0.03	0.07	0.04	0.06	0.04	0.05	0.04	*	*	0.02	0.04	0.03	0.04	*	*	0.02
ALCL	Number of Incidence	21	9	30	31	14	45	22	20	42	23	16	39	28	13	41	27	12	39	30	16	46	31	14	45	21	10	31	16	6	22	23	14	37
	ASR	0.18	0.08	0.13	0.25	0.13	0.19	0.19	0.17	0.18	0.20	0.14	0.17	0.23	0.11	0.17	0.22	0.10	0.16	0.24	0.12	0.18	0.23	0.11	0.17	0.16	0.09	0.13	0.12	0.06	0.09	0.18	0.13	0.15
AITL	Number of Incidence	9	11	20	16	11	27	19	9	28	18	5	23	16	11	27	28	15	43	19	14	33	29	20	49	26	18	44	29	12	41	26	26	52
	ASR	0.08	0.09	0.08	0.12	0.09	0.11	0.15	0.07	0.11	0.14	0.03	0.09	0.13	0.08	0.10	0.21	0.10	0.16	0.13	0.09	0.11	0.20	0.13	0.16	0.18	0.12	0.15	0.19	0.08	0.13	0.17	0.15	0.16
NK/TCL, nasal type	Number of Incidence	20	17	37	24	8	32	32	14	46	29	19	48	43	17	60	34	21	55	46	20	66	40	24	64	31	19	50	40	22	62	48	30	78
	ASR	0.16	0.13	0.15	0.19	0.06	0.13	0.25	0.11	0.18	0.22	0.14	0.18	0.33	0.12	0.22	0.25	0.16	0.20	0.34	0.14	0.24	0.27	0.16	0.21	0.21	0.13	0.17	0.27	0.15	0.21	0.31	0.18	0.24
ATLL	Number of Incidence	*	*	*	*	*	*	*	*	*	*	*	*	*	*	*	*	*	*	*	*	3	*	*	3	*	*	6	*	*	12	*	*	6
	ASR	*	*	*	*	*	*	*	*	*	*	*	*	*	*	*	*	*	*	*	*	0.01	*	*	0.01	*	*	0.02	*	*	0.04	*	*	0.02

*, Not available due to insufficient case numbers.

### 3.2 Distribution of age and sex

**Figure 3:**
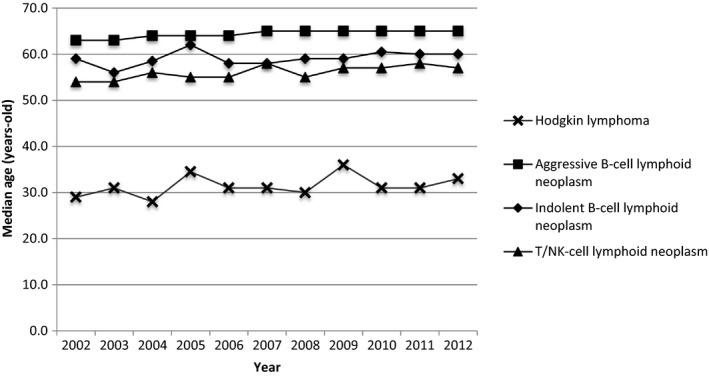
Median age at diagnosis of patients with four major types of lymphoma in Taiwan between 2002 and 2012

**Figure 4:**
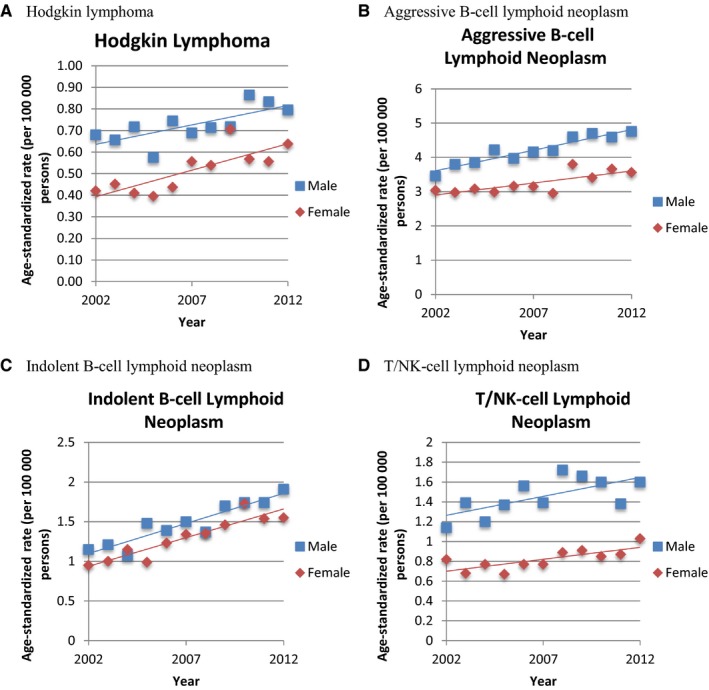
Incidence trends between men and women of four major types of lymphoma in Taiwan between 2002 and 2012 (A) Hodgkin lymphoma, (B) aggressive B‐cell lymphoid neoplasm, (C) indolent B‐cell lymphoid neoplasm, and (D) T/NK‐cell lymphoid neoplasm

NHL tended to occur at older ages than HL. Among NHL patients, the median age at diagnosis of aggressive B‐cell lymphoid neoplasm was the eldest (63.0‐65.0 years) followed by that of indolent B‐cell lymphoid neoplasm (56.0‐62.0 years) and then that of T/NK‐cell lymphoid neoplasm (54.0‐58.0 years) during 2002‐2012 (Figure 3). However, there was heterogeneity among the subtypes. Patients with BL (31.0‐56.0 years) and ALCL (37.0‐54.0 years) were younger at diagnosis than the other subtypes (Figure S4). As for the sex distribution, the patients with T/NK‐cell lymphoid neoplasm were the most male predominant (sex ratio: 1.39‐2.07, Figure S5). Detailed sex ratios of 13 subtypes revealed the male predominance in MCL (sex ratio: 2.18‐6.09) and CLL/SLL (sex ratio: 1.55‐2.55), compared with the sex ratios of aggressive B‐cell lymphoid neoplasm and indolent B‐cell lymphoid neoplasm, respectively (Figure S6). The incidence trends concerning four major types of lymphoma during 2002 to 2012 increased gradually and were similar between men and women (Figure 4). Furthermore, the incidences of most lymphoma were higher in males than those in females, especially for aggressive B‐cell lymphoma (male: 3.46‐4.76 per 100 000; female: 2.95‐3.80 per 100 000) and T/NK‐cell lymphoma (male: 1.14‐1.72 per 100 000; female: 0.67‐1.03 per 100 000) in the four major types, and DLBCL (male: 2.95‐3.84 per 100 000; female: 2.57‐3.18 per 100 000) in the 13 subtypes (Table 2, Figures 4 and S7).

### 3.3 International comparison

**Figure 5:**
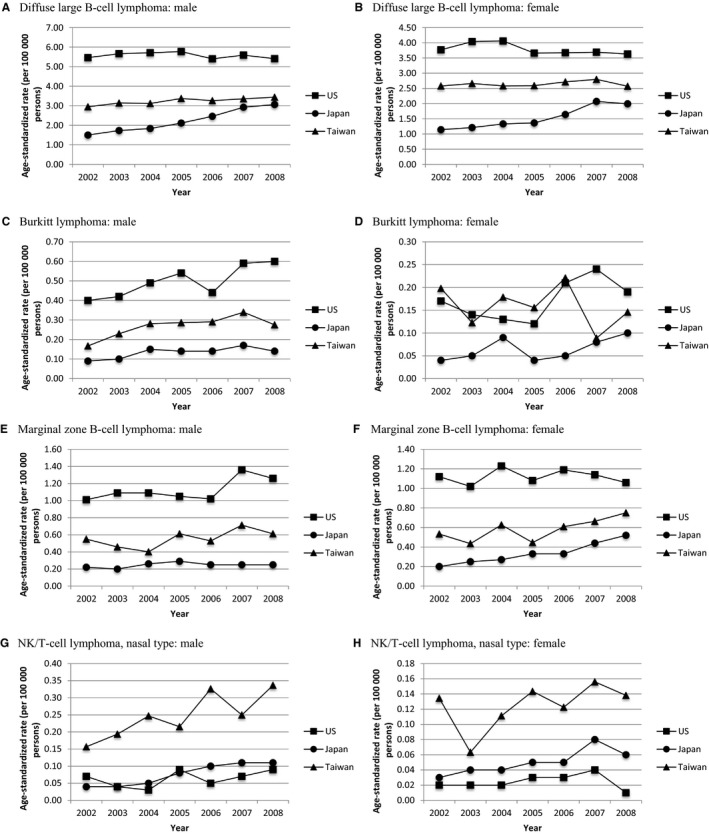
Age‐standardized rates of DLBCL, BL, MZBCL, and NK/TCL, nasal type, in American, Japanese, and Taiwanese males and females between 2002 and 2008 (A) Diffuse large B‐cell lymphoma: male, (B) diffuse large B‐cell lymphoma: female, (C) Burkitt lymphoma: male, (D) Burkitt lymphoma: female, (E) marginal zone B‐cell lymphoma: male, (F) marginal zone B‐cell lymphoma: female, (G) NK/T‐cell lymphoma, nasal type: male, and (H) NK/T‐cell lymphoma, nasal type: female

Comparisons of subtype‐specific incidence rates of lymphoid malignancies among the United States, Japan, and Taiwan are depicted in Figures 5 and S8, and detailed data are presented in Table S1. Notably, during 2002 to 2008, the ASRs of Hodgkin lymphoma in Taiwan (male: 0.57‐0.74 per 100 000, female: 0.40‐0.56 per 100 000) were obviously lower than that in the United States (male: 2.72‐3.17 per 100 000, female: 2.17‐2.60 per 100 000) and comparable to that in Japan (male: 0.38‐0.59 per 100 000, female: 0.24‐0.47 per 100 000) in both sex. The ASRs of aggressive and indolent B‐cell lymphoid neoplasms were distinctly different among the United States, Taiwan, and Japan. In patients with DLBCL (Table 2, Figure 5A,B) and MZBCL (Table 2, Figure 5E,F), and in male patients with BL (Table 2 and Figure 5C), the incidences were highest in the US population, lowest in the Japanese population, and intermediate in the Taiwanese population. In contrast, the ASRs were divergent in NK/TCL, nasal type (Table 2, Figure 5G,H). The ASRs of NK/TCL were highest in the Taiwanese population and lowest in the US population.

## 4 DISCUSSION

To the best of our knowledge, this population‐based study is the largest and the most comprehensive epidemiologic study to provide information on subtype‐specific epidemiology of lymphoid malignancies in the Taiwanese population, and it shows the differences in incidences among Taiwan, Japan, and the United States. Our study has the merits of using the population‐based data, which fills the knowledge gap that many of the existing studies are limited to single‐center studies and thought to be unrepresentative.^8^, ^14^, ^15^ In addition, using the year 2000 world standard population for incidence, adjustment made us able to compare our findings to those in Japan and the United States from the previous studies.^7^

In our study, the ratio of HL and NHL is consistent with the previous smaller‐scale study conducted in Taiwan.^8^ HL and NHL accounted for 6.09% and 93.32% of all lymphoid malignancies in the previous study,^8^ and our study obtained the similar results (HL, 7.9%; and NHL, 92.1%). Compared with studies from other countries, NHL tended to occur more frequently in both Eastern and Western countries although the percentages of HL are higher in Western countries.^7^, ^8^, ^14^, ^15^, ^16^, ^17^ In the United States and the United Kingdom (UK), HL constituted 9.96% and 14.41% of patients with lymphoid neoplasms.^16^, ^17^ In Eastern countries, by contrast, HL accounted for 4.12%, 5.9%, 8.60%, and 7.43% of patients with lymphoid neoplasms in South Korea, Japan, China, and Taiwan (our study), respectively.^7^, ^14^, ^15^

Because NHL is composed of different types of lymphoma, we further make the comparisons between our results and the previously published one from other countries. B‐cell lymphoma (BCL) accounts for the majority of NHL in both Western and Eastern countries.^7^, ^8^, ^14^, ^15^, ^16^, ^17^ In the United States and UK, BCL composes of 93.37% and 93.79% of NHL cases, respectively.^16^, ^17^ However, the percentages of BCL are slightly lower and are 81.60%, 77.05%, 71.05%, and 83.67% of NHL patients in South Korea, Japan, China, and Taiwan (our study).^7^, ^14^, ^15^ In detail, the majority of BCL cases was DLBCL in both Eastern and Western countries, and the percentages were variable (38.92% in the United States,^18^ 43.24% in the UK,^17^ 45.8% in the Central and South America,^19^ 47.75% in South Korea,^15^ 62.48% in Japan,^7^ 55.78% in Mainland China,^14^ and 57.04% in Taiwan, our study). The second and third frequent types of BCL are FL and MZBCL in Japan and Taiwan (our study). FL and MZBCL accounted for 18.60% and 9.93% of BCL patients in Japan,^7^ and 12.22% and 11.80% in Taiwan. However, the percentage of FL is lower in Mainland China (4.48%)^14^ and South Korea (2.68%).^15^ Why the frequencies of FL differ among Asian countries remains uncertain. The previous study suggests several potential risk factors, such as diet modernization, inoculation rate of influenza vaccination, single nucleotide polymorphisms (SNPs) within the major histocompatibility complex (MHC) region, and environmental factors.^20^ Compared to our study, MZBCL (MALT included) in South Korea is the second majority of BCL, and it composed 19.45% of patients diagnosed with BCL.^15^ *Helicobacter pylori* infection is related to MALT, and *H. pylori* irradiation is the frontline treatment for the patients with limited stage MALT.^21^ Therefore, higher proportion of MZBCL in South Korea may be related to the high prevalence of *H. pylori* and endoscopy screening policy for gastric cancer.^22^, ^23^

About T‐cell lymphoma (TCL), the incidence rates are higher in Eastern countries than those in Western countries.^7^, ^8^, ^14^, ^15^, ^16^, ^17^ In the United States and the UK, TCL accounts for 6.63% and 6.21% of NHL, respectively,^16^, ^17^ whereas the incidences of TCL in South Korea, Japan, Mainland China, and Taiwan (our study) are threefold to fourfold higher (17.16%, 19.98%, 28.95%, and 16.33% of NHL patients, respectively).^7^, ^14^, ^15^ Nevertheless, the frequencies of TCL subtypes vary among Asian countries. The most frequent type in Japan is ATLL (45.86% of TCL),^7^ but PTCL‐NOS in South Korea (31.6%) and Taiwan (31.31%),^15^ and NK/TCL in Mainland China (47.04%).^14^ In South Korea, NK/TCL is the second common TCL and constitutes 30.9% of TCL patients.^15^ The disparity in the distribution of TCL subtypes among countries may be attributable to different lifestyles, environmental factors, and genetic polymorphisms.^24^, ^25^ In addition, viral infections also play a pivotal role in TCL, such as EBV infections in NK/TCL, nasal type, and HTLV‐1 infections in ATLL. High prevalence of EBV infection in Asian countries and high HTLV‐1 carrier rate in south Japan contribute to the relatively high incidence of NK/TCL and ATLL in these area.^4^, ^5^ Unlike southern Japan, in which the HTLV‐1 prevalence was reported to be the highest in the world (more than 10%), the HTLV‐1 prevalence was reported to be between 0.1 and 1% in Taiwan.^26^ Another study also reported that the prevalence of HTLV‐1 in Japan was between 1 080 000 and 1 300 000/127 368 088 persons and that in Taiwan was between 10 000 and 30 000/23 113 901 persons.^27^ These may explain the differences of prevalence of ATLL in Japan and Taiwan.

In addition to disease incidences, we further compare age‐standardized rates (ASRs) of lymphoid malignancies in Taiwan with those in Japan and the United States, and all adjustments are made according to the 2000 world standard population as defined by the World Health Organization.^7^ In most lymphoid malignances, the ASRs are the highest in the United States followed by those in Taiwan and then in Japan, especially in HL, aggressive B‐cell lymphoma, and indolent B‐cell lymphoma. In TCL, however, except for MF, CTCL, and ALCL, ASRs in Taiwan were mostly the highest in PTCL‐NOS, NK/TCL, and AITL during 2002 to 2008. As for comparisons with Hong Kong (2001‐2010), South Korea (1999‐2012), and Surveillance, Epidemiology, and End Results Program (SEER) data in Asian Americans (2001‐2010), notably, the ASR of HL in Taiwan (0.49‐0.72 per 100 000 persons) was lower than that in SEER Asian Americans (1.28 per 100 000 persons) but comparable to that in Hong Kong (0.75 per 100 000 persons) and higher than that in South Korea (0.35 per 100 000 persons). And likewise, ASRs of BCL among SEER Asian Americans, Taiwan, Hong Kong, and South Korea, including DLBCL, FL, MCL, BL, and CLL/SLL, showed the same pattern, in which ASRs of SEER Asian Americans were the highest followed by those of Taiwan and Hong Kong, and then those of South Korea. As for TCL, ASR of PTCL‐NOS in Taiwan (0.28‐0.40 per 100 000 persons) was higher or comparable to that in SEER Asian Americans (0.28 per 100 000 persons), in Hong Kong (0.27 per 100 000 persons), and in South Korea (0.26 per 100 000 persons). For ASR of NK/TCL, that in Taiwan (0.13‐0.24 per 100 000 persons) was comparable to that in Hong Kong (0.25 per 100 000 persons) and in South Korea (0.22 per 100 000 persons), and was obviously higher than that in SEER Asian Americans (0.12 per 100 000 persons).^6^, ^9^ The differences in ASRs of lymphoid malignancies are not only observed between Eastern and Western countries but also among Asians living in diverse countries. This observation strengthens the assumption that the etiology of lymphoid malignancies consists of genetics, environmental factors, and lifestyles.

As for subtype‐specific sex ratio of lymphoid malignancies in Taiwan, male predominance is most obvious in MCL patients (sex ratio, 2.18‐6.09), which is consistent with the results found in Hong Kong and in the United States (standardized rate ratio (SRR) in Hong Kong, 4.3; incidence rate and rate ratio (IRR) in the United States, 3.07).^6^, ^28^ However, etiology of the male predominance in MCL still remains unclear. Interestingly, despite the male predominance noticed in most subtypes of lymphoma, no such phenomenon was observed in DLBCL (sex ratio, 1.11‐1.29), MZBCL (sex ratio, 0.64‐1.28), and FL (sex ratio, 0.87‐1.44). These findings are consistent with the results in Hong Kong, where SRRs of DLBCL, MZBCL, and FL were 1.3, 1.1, and 1.1, respectively.^6^ As for the United States, though IRRs of MZBCL (1.05) and FL (1.18) were the lowest among all subtypes as well, DLBCL was a bit more male‐predominated than Taiwan and Hong Kong instead with IRR of 1.49.^28^ Further research concerning subtype‐specific etiology of lymphoma is still urgently required to elucidate the sex difference.

While we provide tremendous epidemiologic information of lymphoma, the present study has some limitations. First, the incidence of some TCL in Taiwan was extremely low, such as MF, CTCL, and ATLL. Therefore, it should be very cautious when making comparisons with the incidences in other countries. Second, the subtype‐specific incidences of lymphoma in the South Korea population were adjusted according to Segi world population, which is different from the standard applied in our study.^9^ The slight discrepancies may therefore be arisen when we try to make the comparisons with the data in South Korea. Nevertheless, there are still merits of our study. Using population‐based data source, our results are representative enough of the Taiwanese population. Moreover, adjustments made on incidence rates according to the 2000 world standard population let us be able to evaluate the dissimilarity existing among countries. In this way, our data are worth helping unveil the rather mysterious etiology of lymphoma.

Conclusively, our study is the first population‐based study conducted in Taiwan aiming to investigate subtype‐specific epidemiology of lymphoma. Most of the incidence rates of lymphoid malignancies in Taiwan are lower than those in the United States, and higher than or comparable to those in Japan except that the ASRs of some TCL are the highest, including PTCL/NOS, AITL, and NK/TCL. The researches regarding the subtype‐specific epidemiology of lymphoid malignancies in different countries or regions are crucial to elucidate the etiologies of lymphoma. Further investigations about the subtype‐specific survival of lymphoid malignancies in Taiwan are required to clarify the natural history of lymphoma and the impact of ever‐advancing therapies.

## CONFLICT OF INTERESTS

KBS, LCY, CHM, and HFY received a research grant sponsored by Roche Products Ltd. (Taiwan).
