# Supplementary material for: Subtype‐specific epidemiology of lymphoid malignancies in Taiwan compared to Japan and the United States, 2002‐2012
Source: Cancer Med. 2018 Oct 9;7(11):5820–31. doi: 10.1002/cam4.1762 (PMC6246924; doi:10.1002/cam4.1762)
Supplement: Supplementary file 1 [file CAM4-7-5820-s001.pdf]

Figure S1. Crude incidence rates of 13 subtypes of lymphoma in Taiwan between the years 2002-2012

A. Aggressive B-cell lymphoid neoplasm (Logarithm)

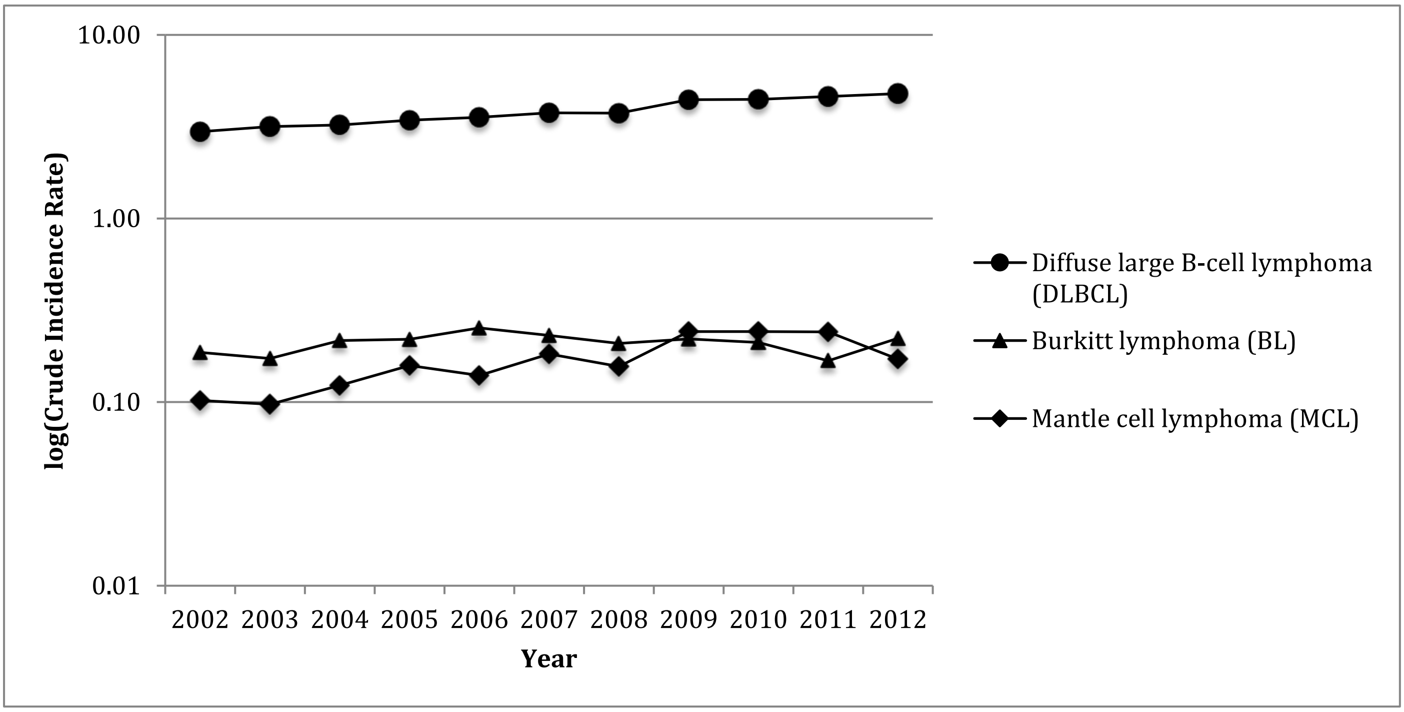

B. Indolent B-cell lymphoid neoplasm

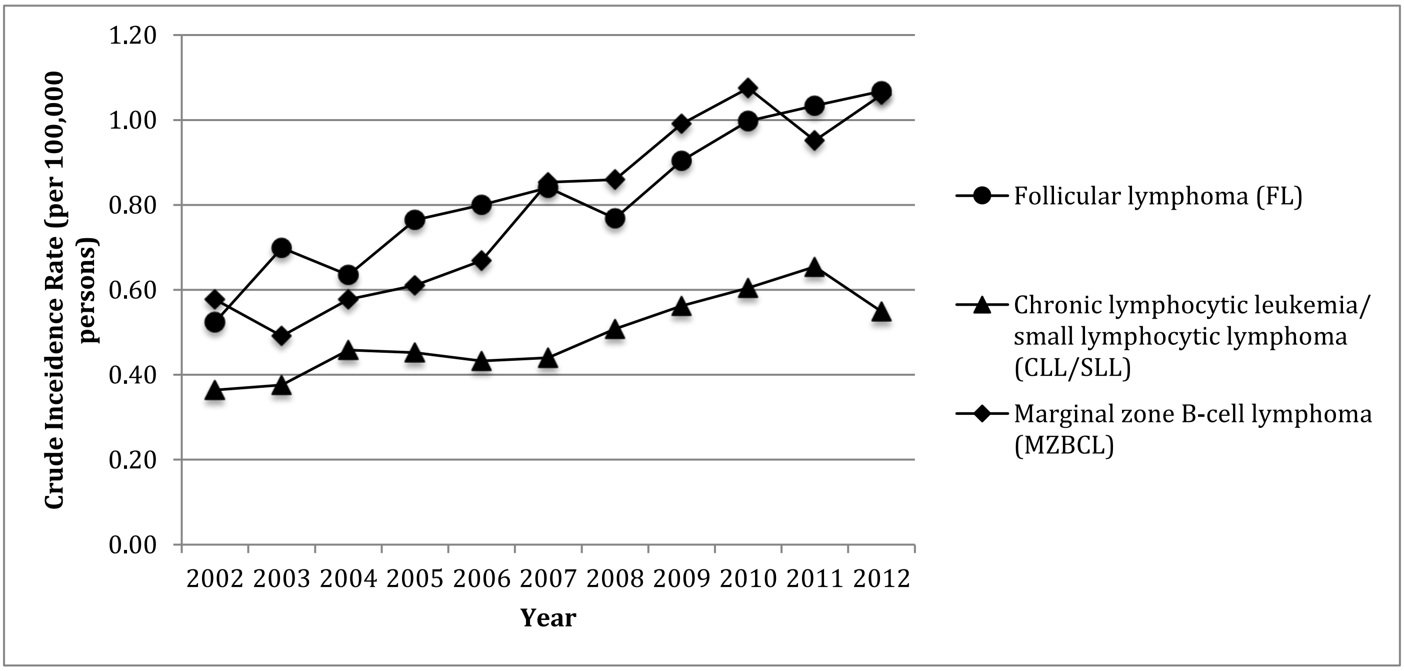

C. T/NK-cell lymphoid neoplasm

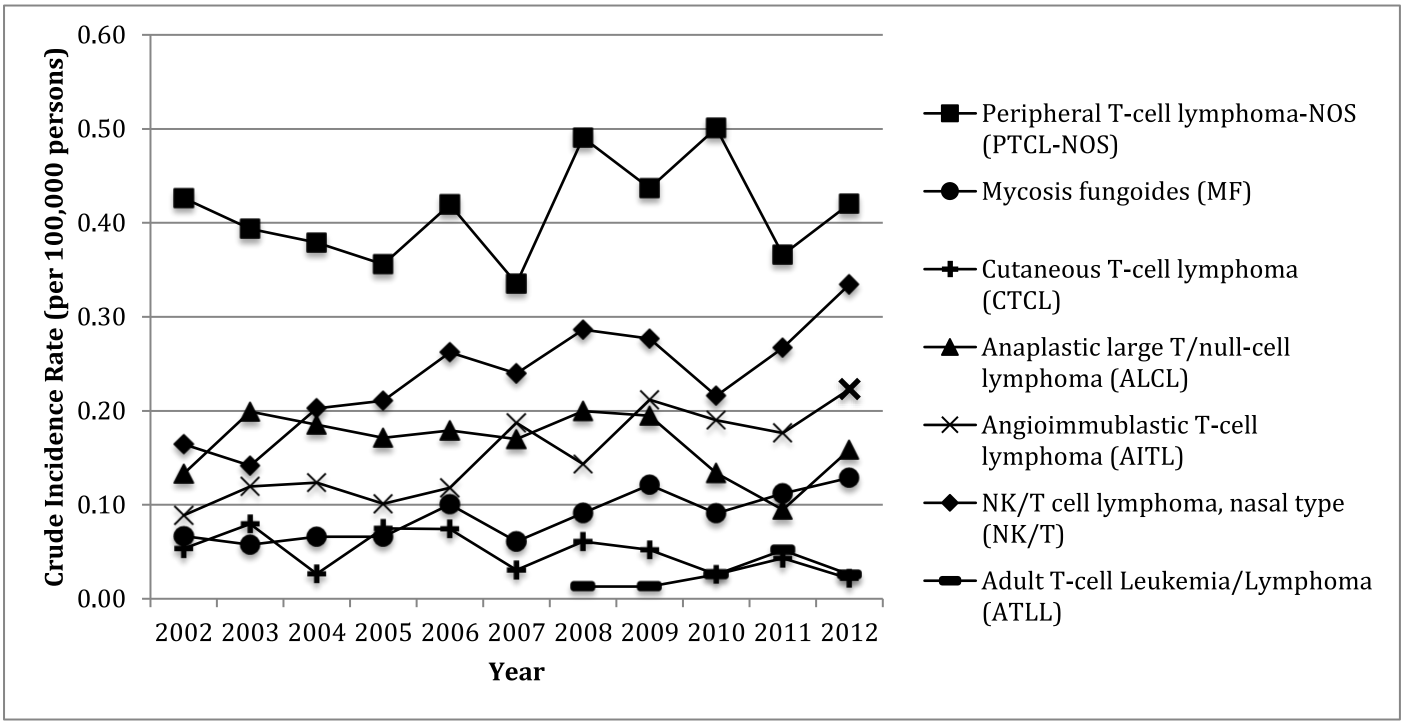

Figure S2. Age-standardized rates of 13 subtypes of lymphoma in Taiwan between the years 2002-2012

A. Aggressive B-cell lymphoid neoplasm (Logarithm)

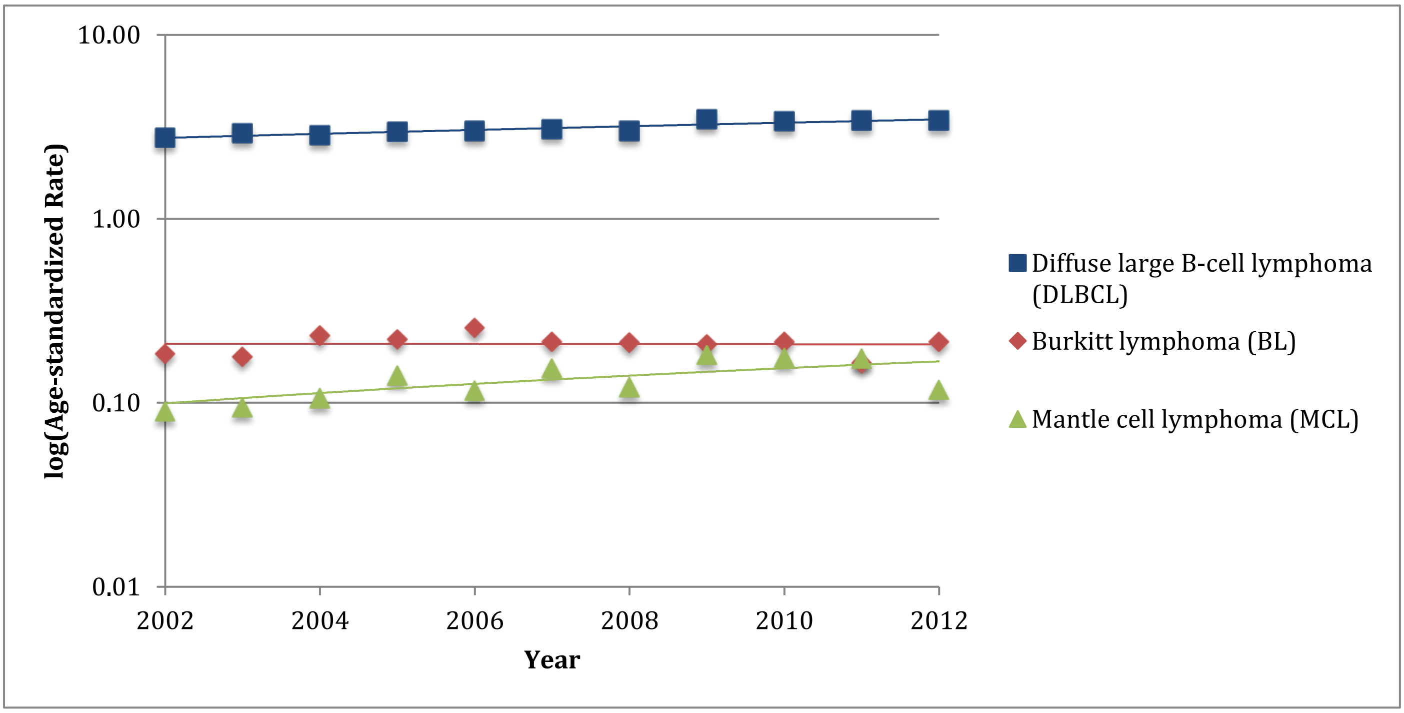

B. Indolent B-cell lymphoid neoplasm

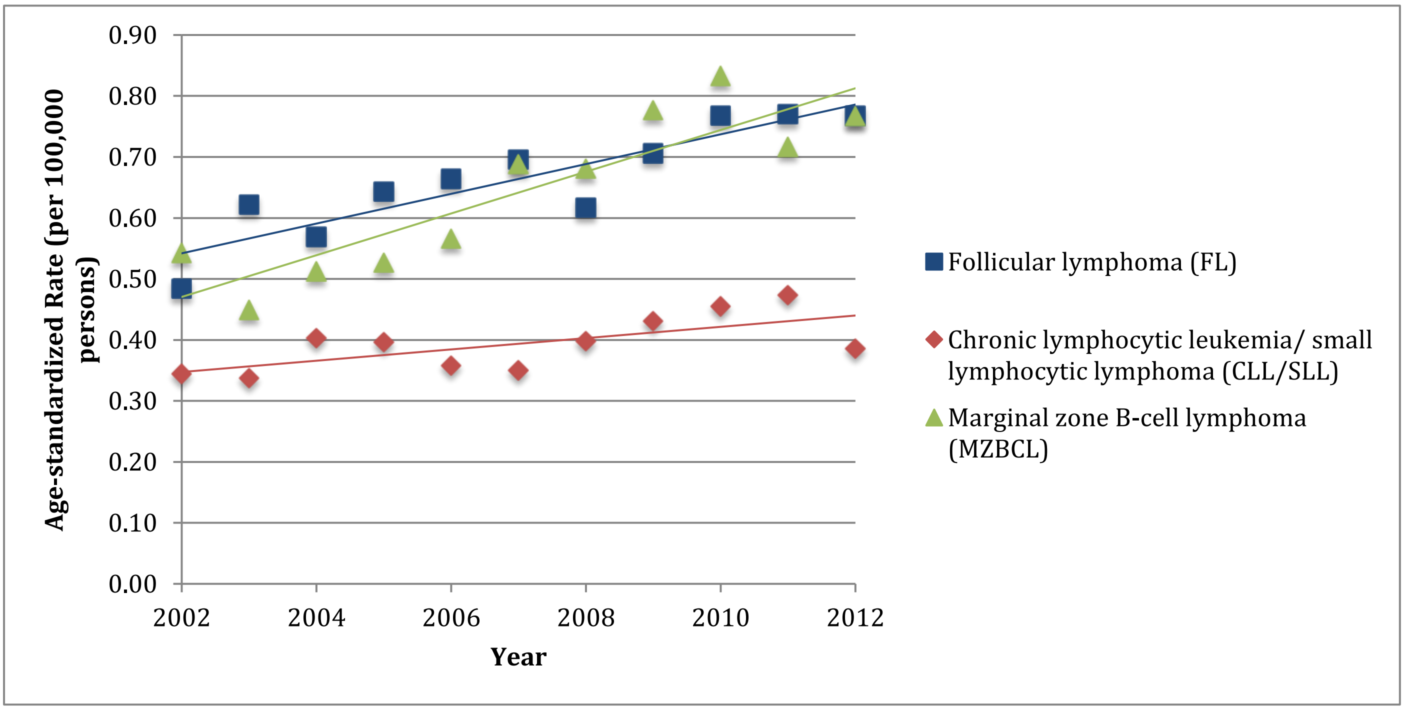

C. T/NK-cell lymphoid neoplasm

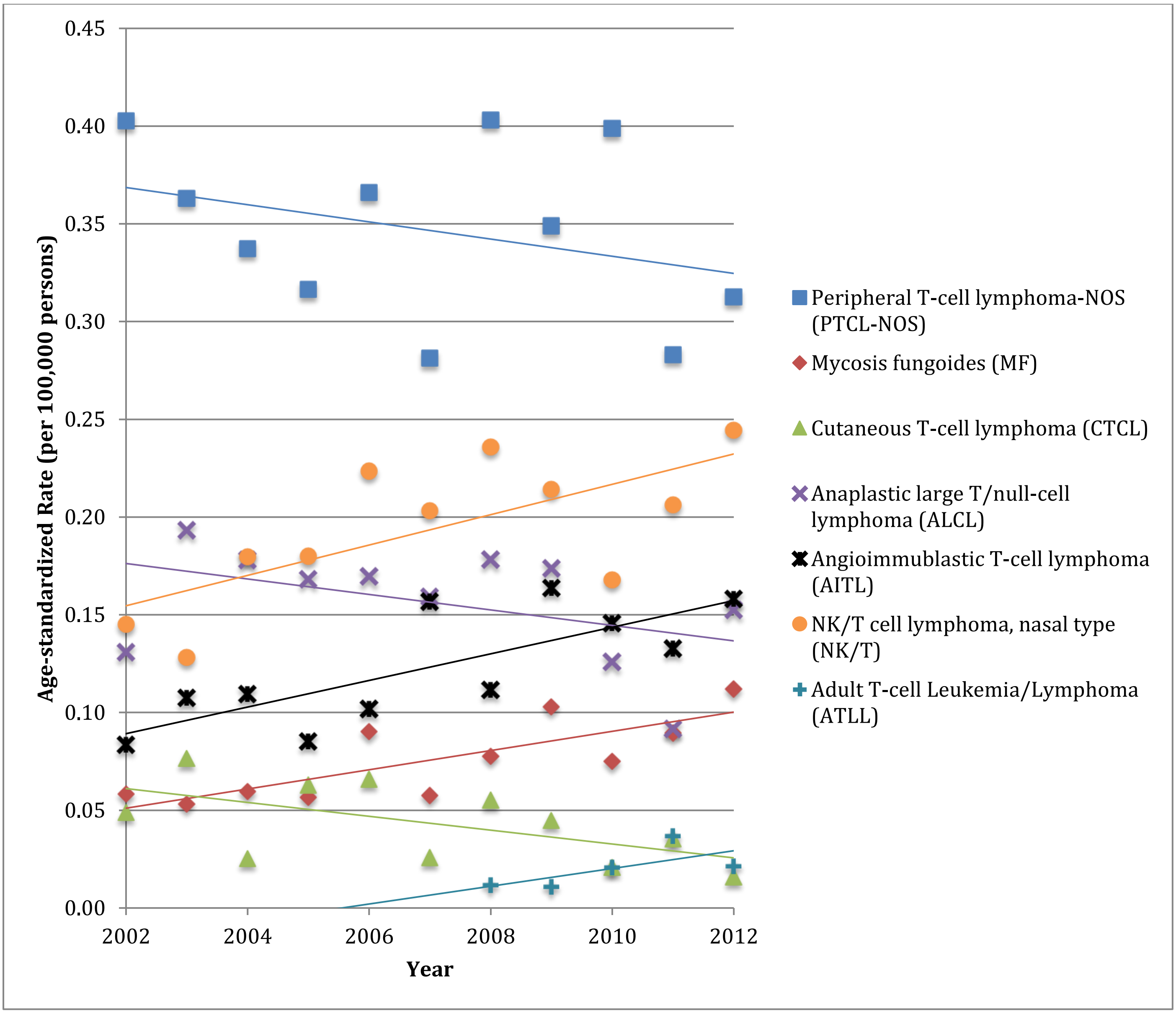

Figure S3. Percentages of four major types of lymphoma in Taiwan between the years 2002-2012

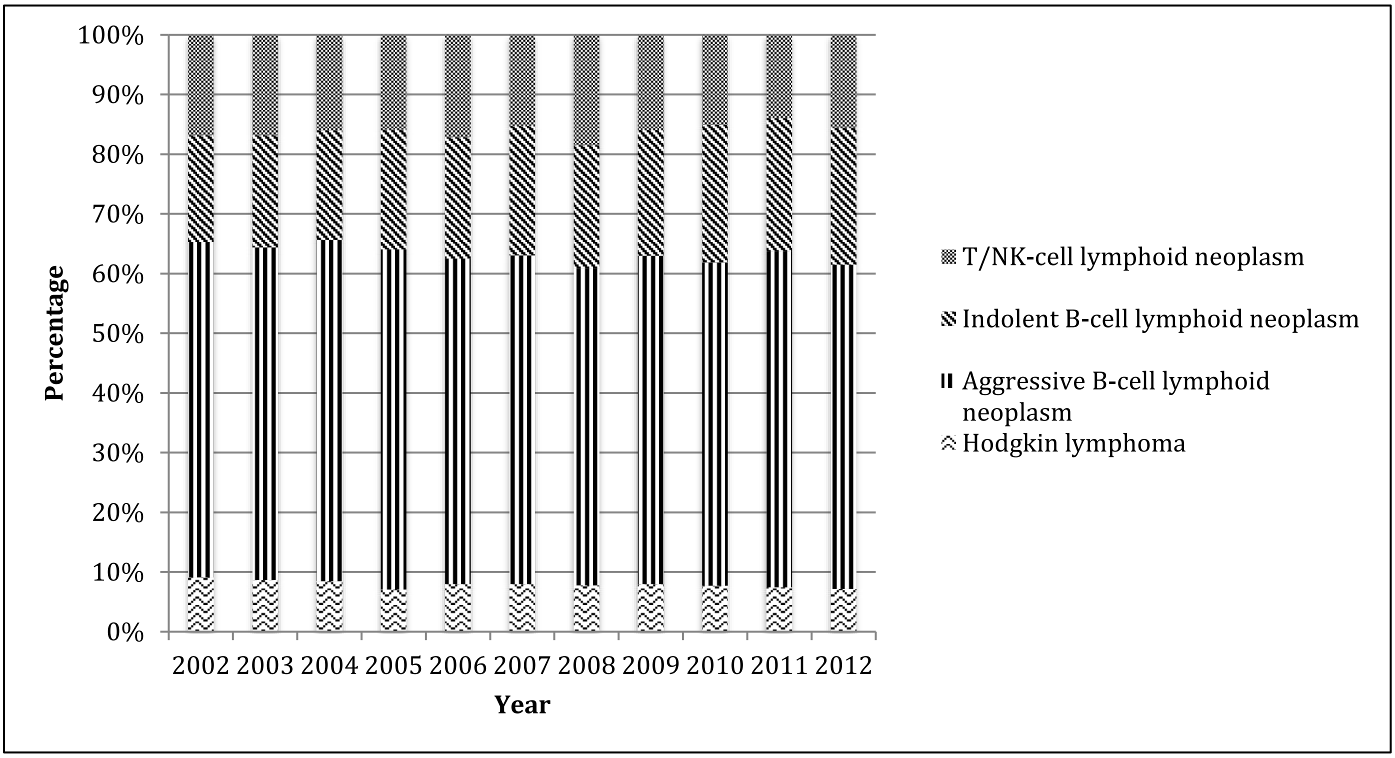

Figure S4. Median age at diagnosis of 13 subtypes of lymphoma in Taiwan between the years 2002-2012

A. Aggressive B-cell lymphoid neoplasm

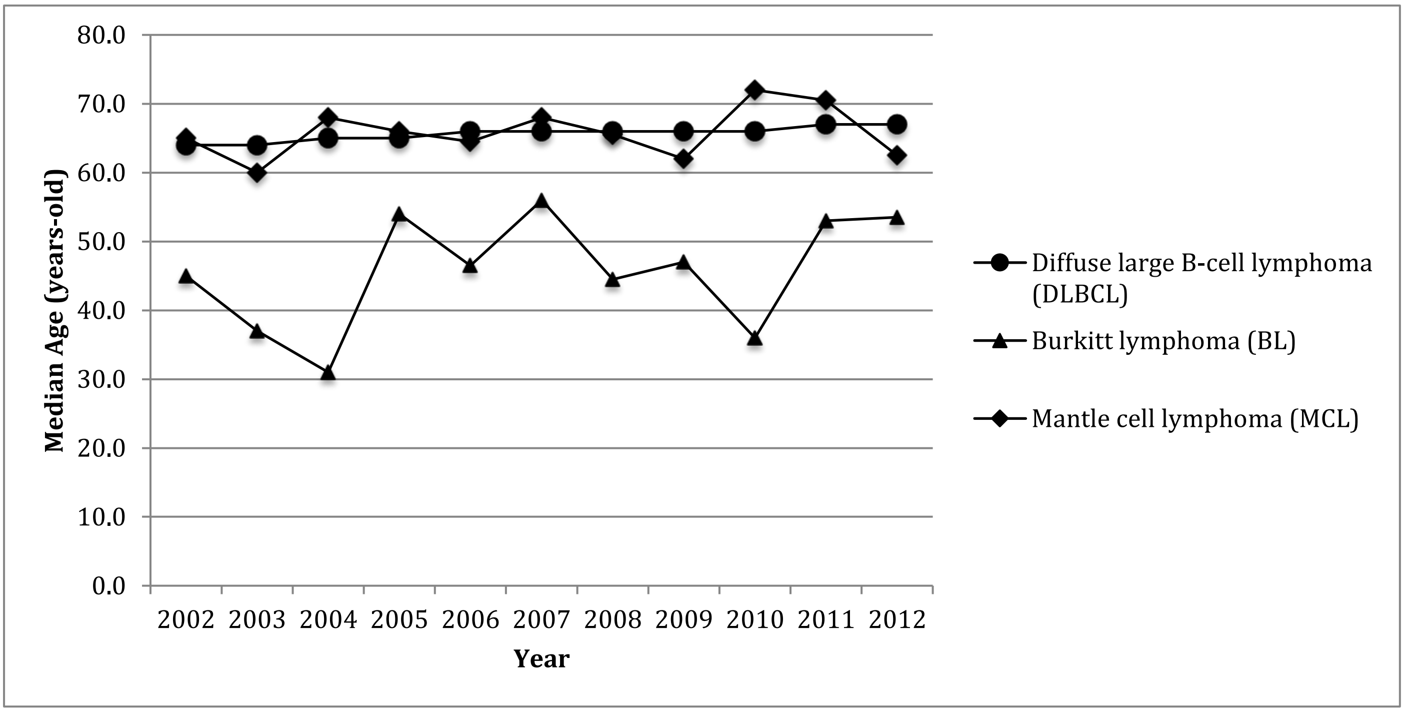

B. Indolent B-cell lymphoid neoplasm

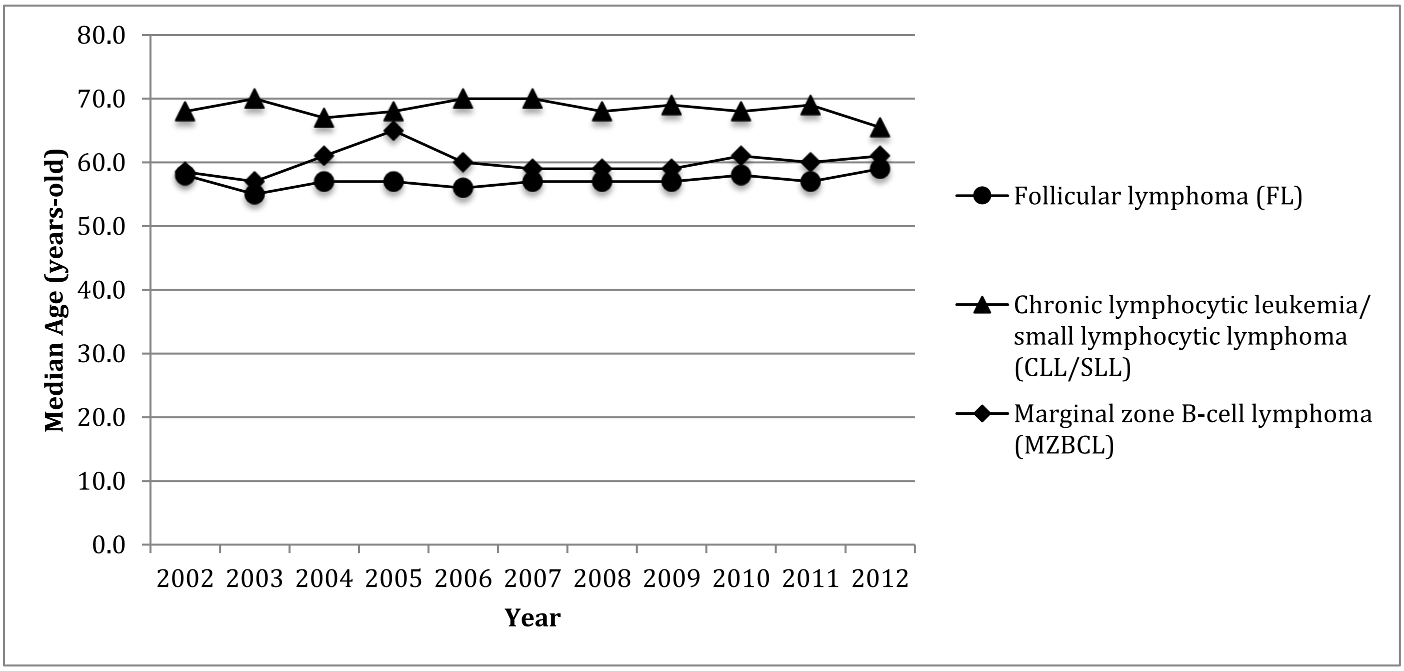

C. T/NK-cell lymphoid neoplasm

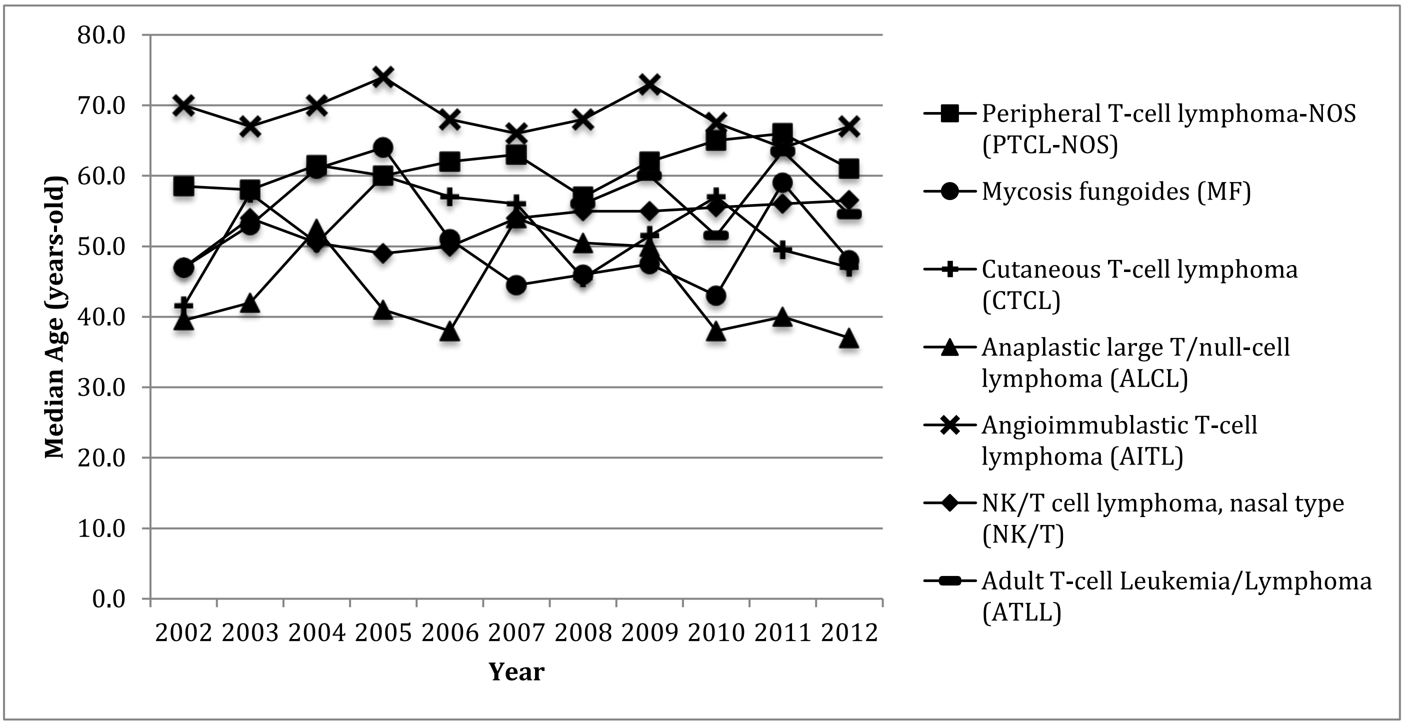

*Lack of data in ATLL during 2002-2007 due to insufficient cases defined as less than 3

Figure S5. Sex ratio of patients with four major types of lymphoma in Taiwan between the years 2002-2012

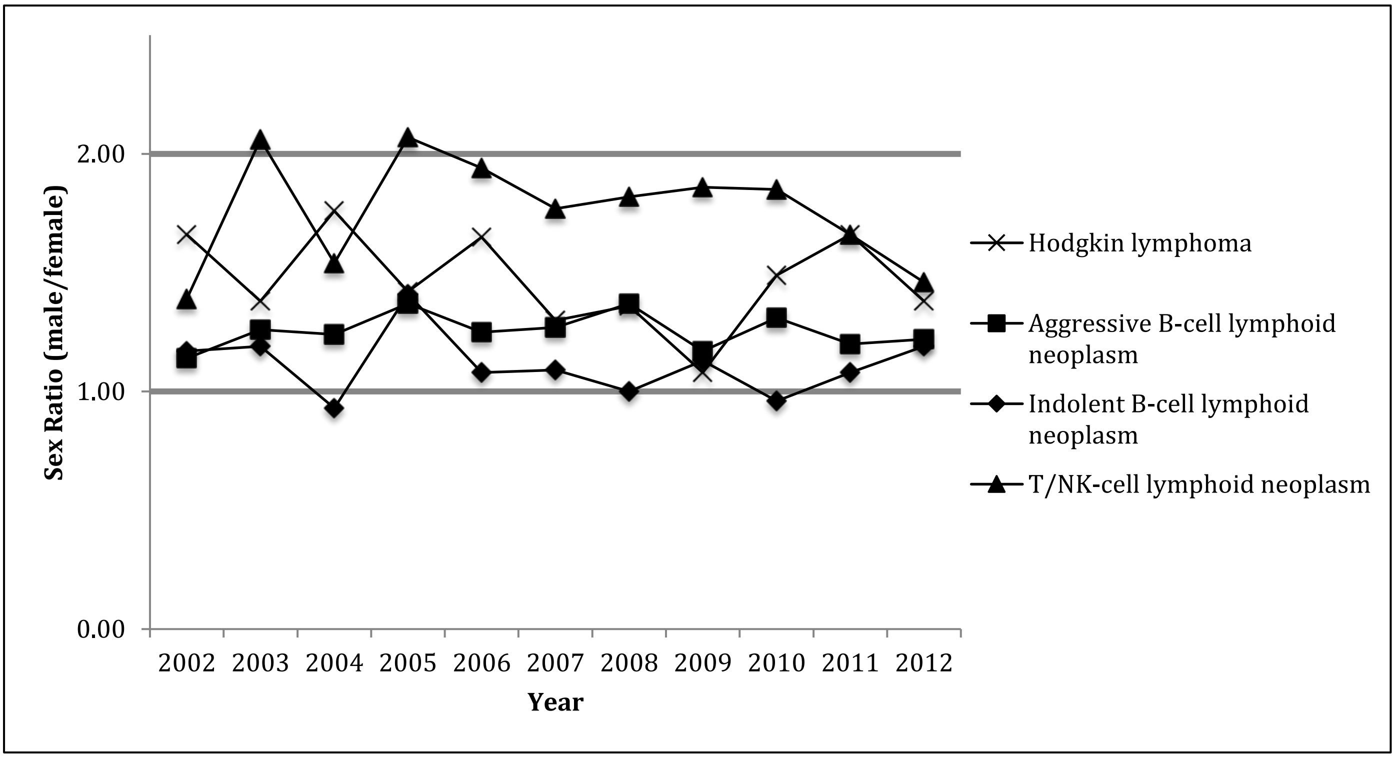

Figure S6. Sex ratios of 13 subtypes of lymphoma in Taiwan between the years 2002-2012

A. Aggressive B-cell lymphoid neoplasm

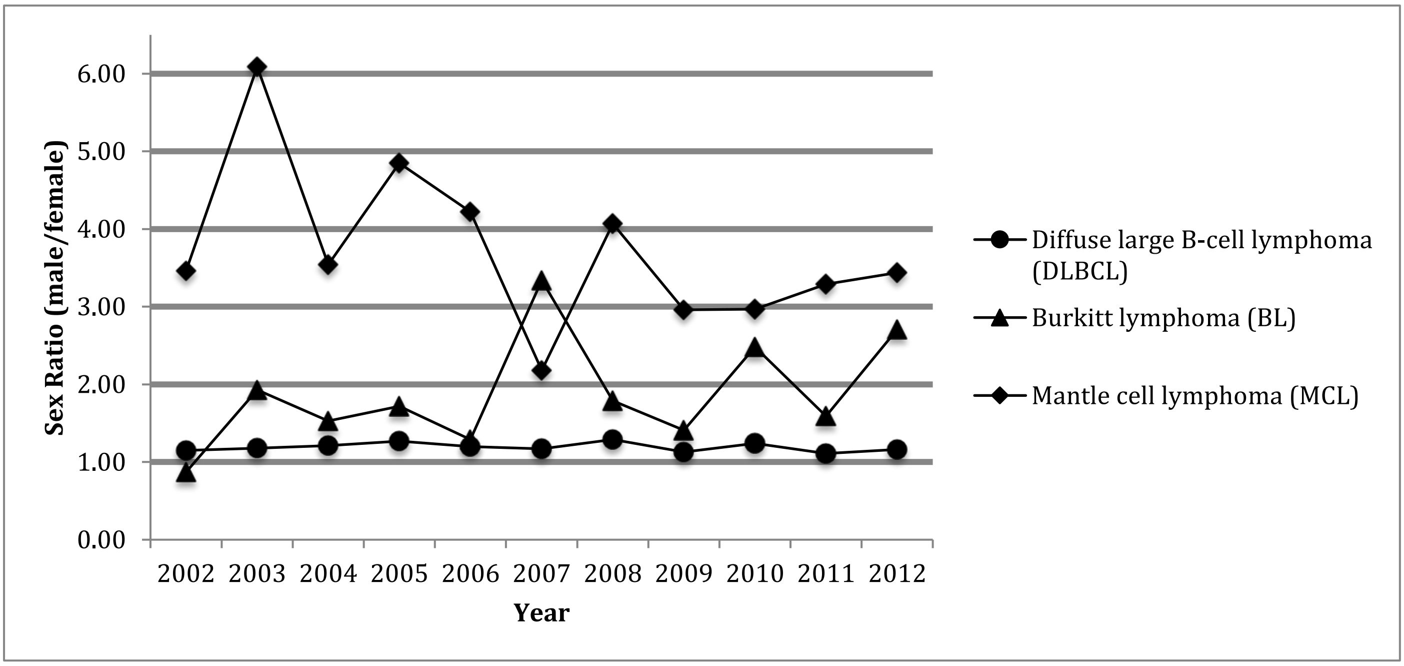

B. Indolent B-cell lymphoid neoplasm

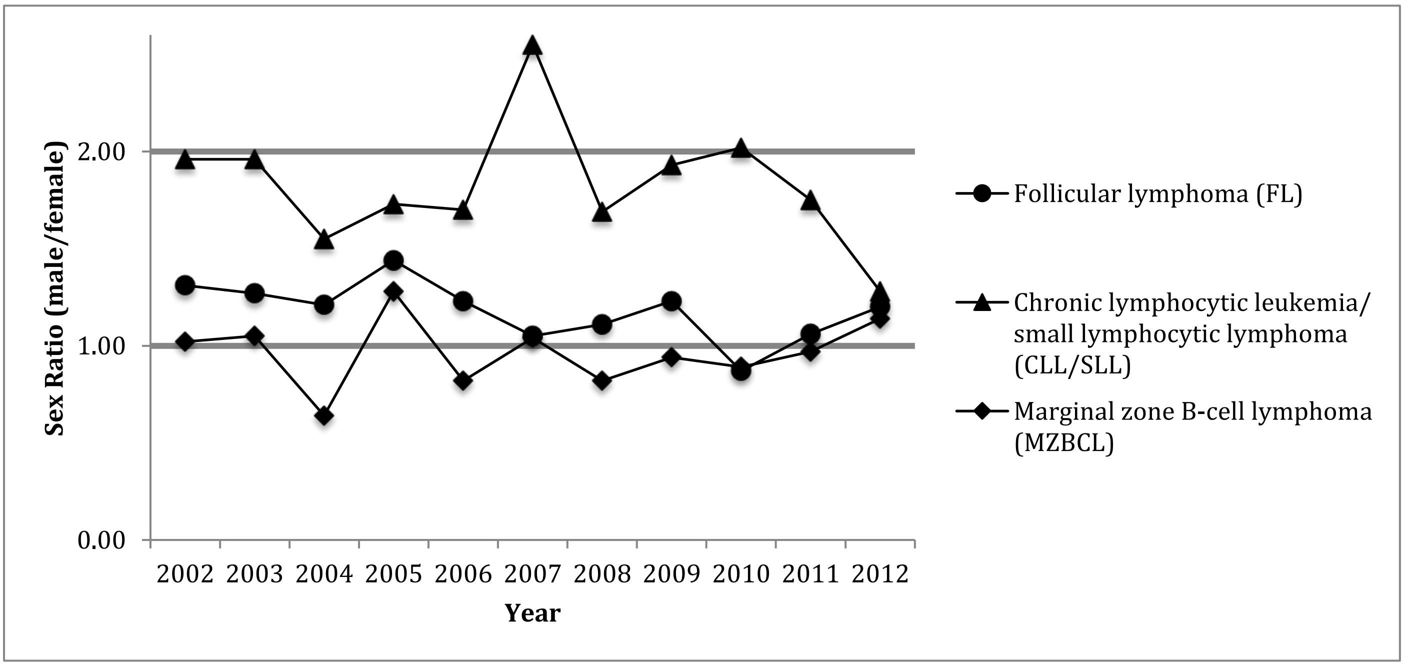

C. T/NK-cell lymphoid neoplasm

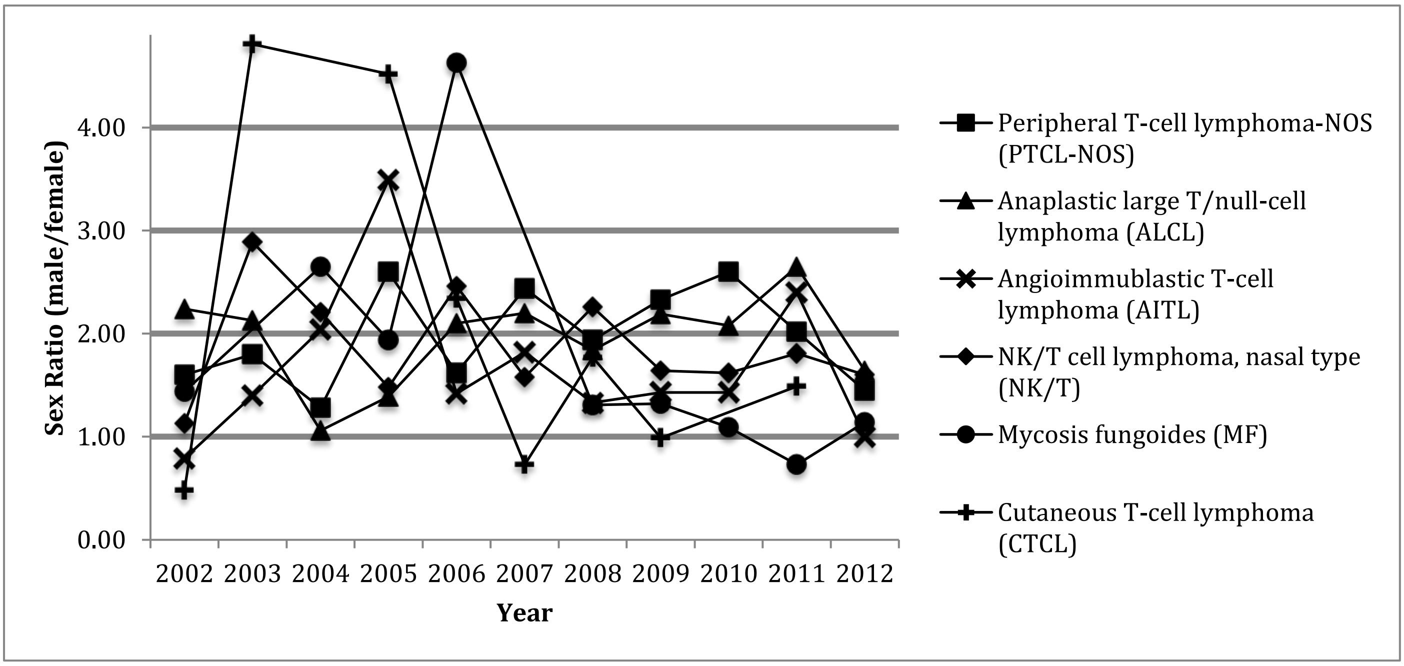

*Adult T-cell Leukemia/Lymphoma (ATLL) is not shown in this figure due to insufficient cases (< 3).

*Lack of sex ratio data of Mycosis fungoides (MF) in 2003 and 2007 due to insufficient cases (< 3).

*Lack of sex ratio data of Cutaneous T-cell lymphoma (CTCL) in 2004, 2010, and 2012 due to insufficient cases (< 3).

Figure S7. Incidence trends between men and women of 13 subtypes of lymphoma in Taiwan between the years 2002-2012

Diffuse large B-cell lymphoma

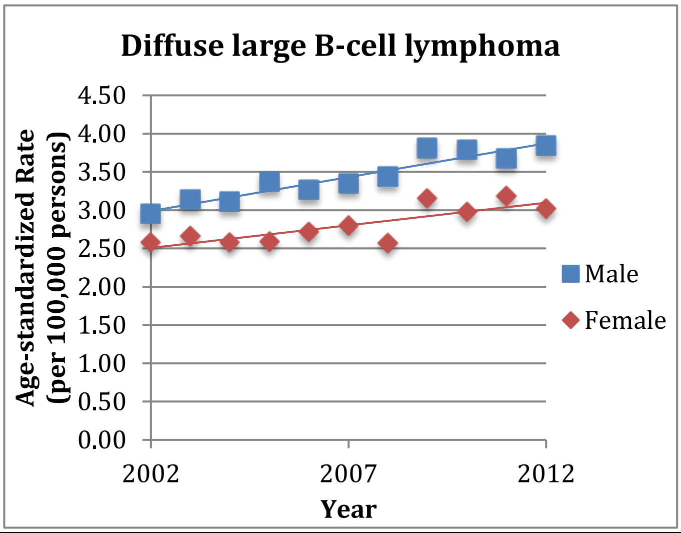

Burkitt lymphoma

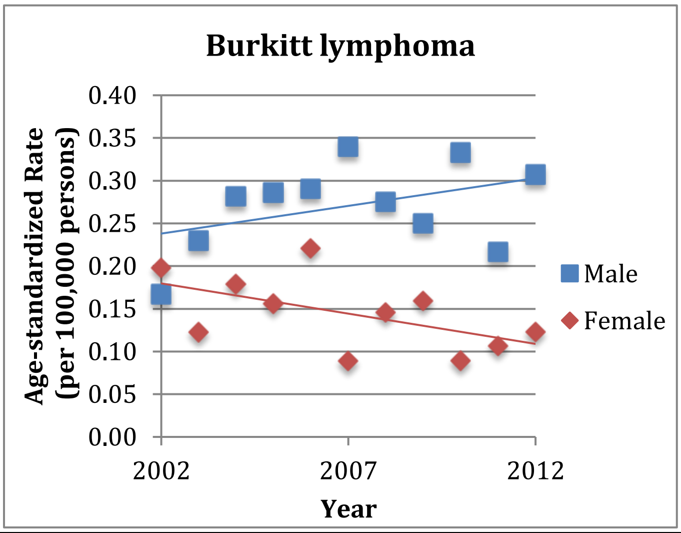

Mantle cell lymphoma

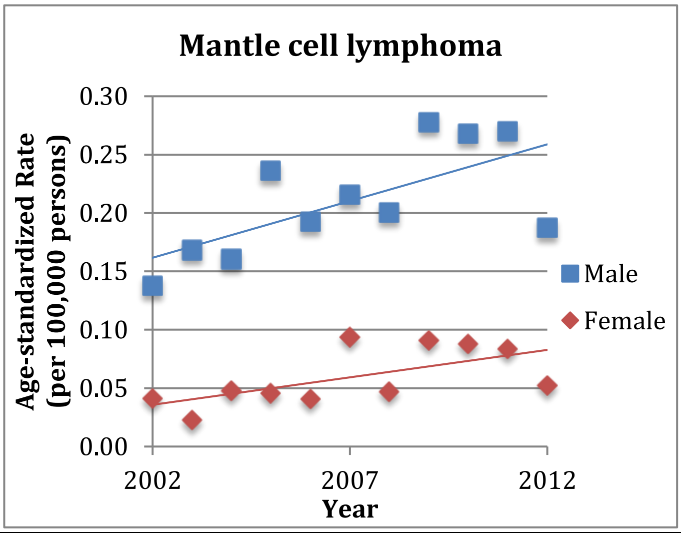

Follicular lymphoma

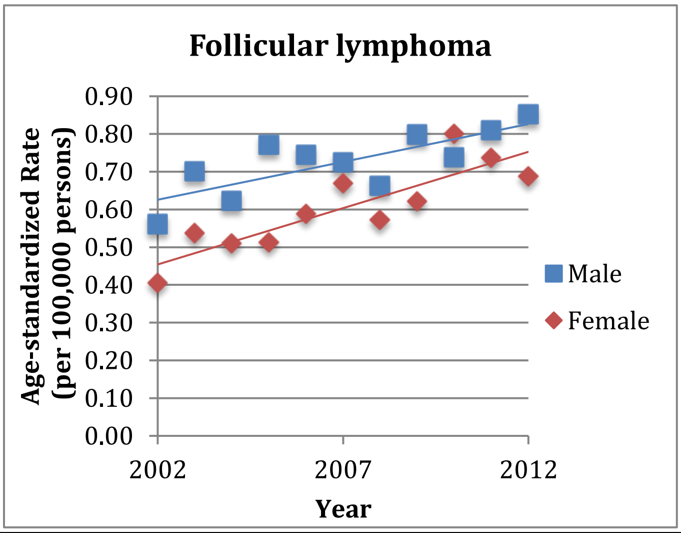

Chronic lymphocytic leukemia/ small lymphocytic lymphoma

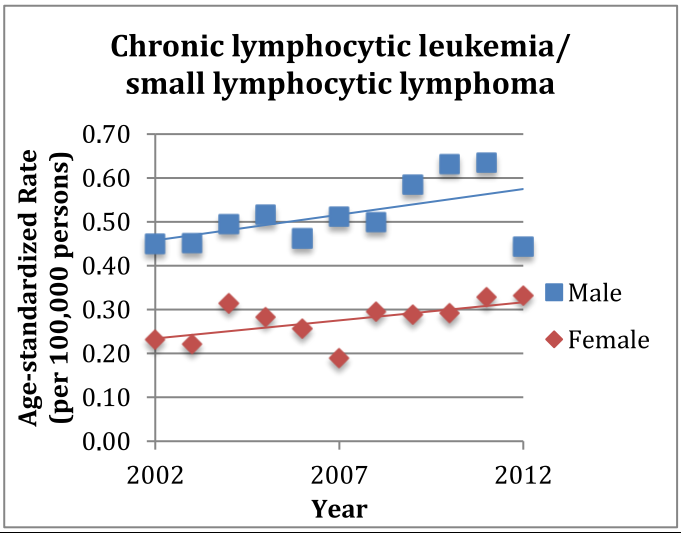

Marginal zone B-cell lymphoma

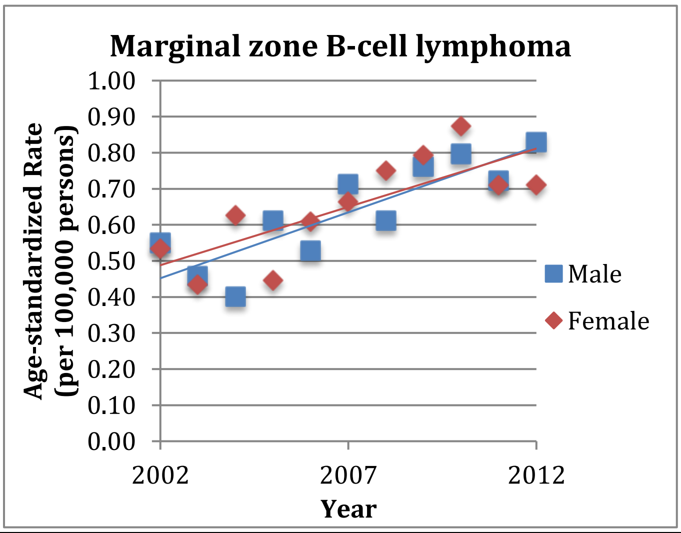

Figure S7. Incidence trends between men and women of 13 subtypes of lymphoma in Taiwan between the years 2002-2012 (*continued*)

Peripheral T-cell lymphoma-NOS

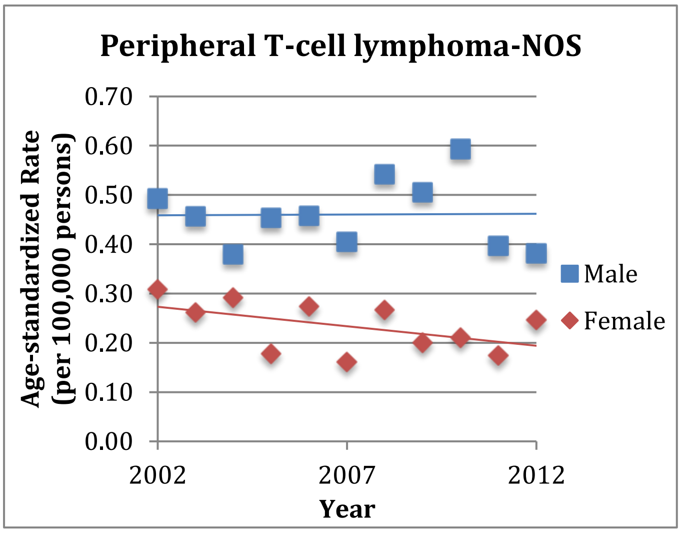

Mycosis fungoides

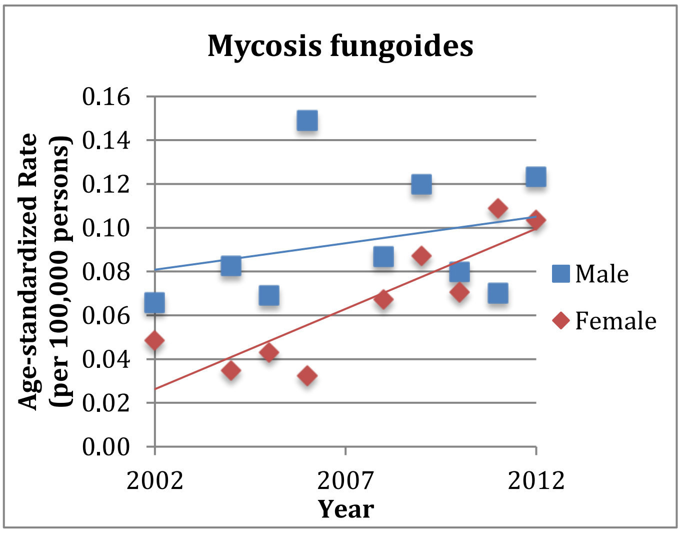

Cutaneous T-cell lymphoma

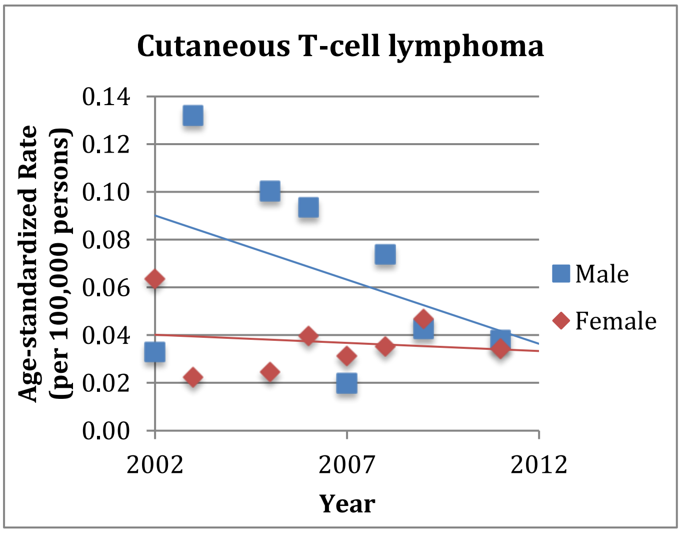

Anaplastic large T/null-cell lymphoma

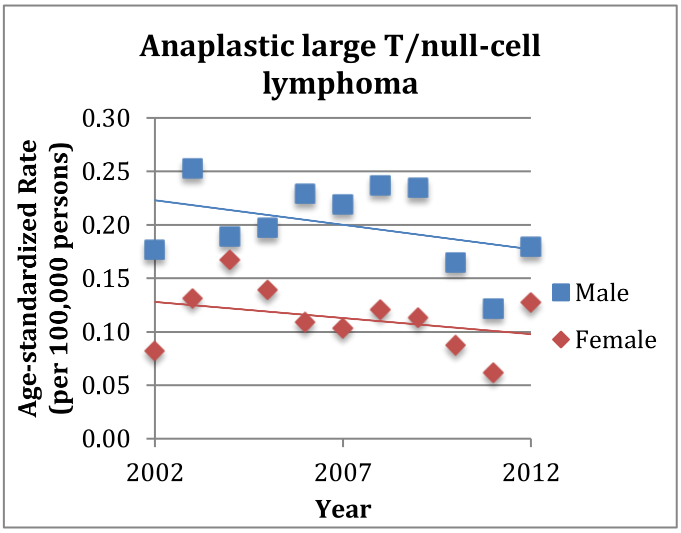

Angioimmublastic T-cell lymphoma

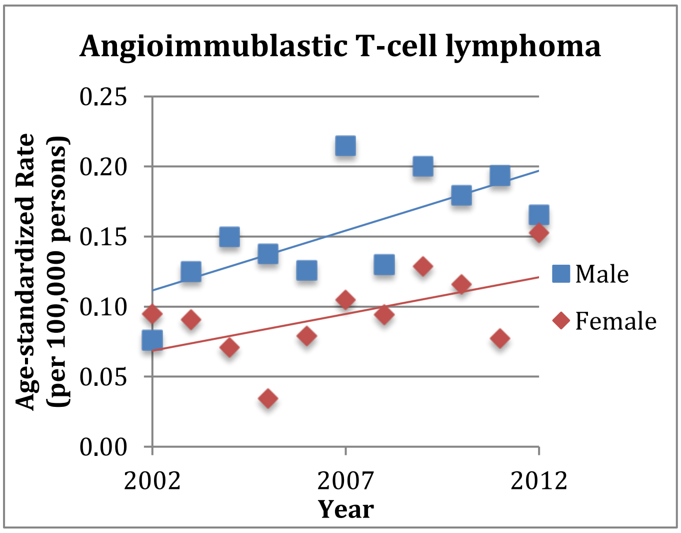

NK/T-cell lymphoma, nasal type

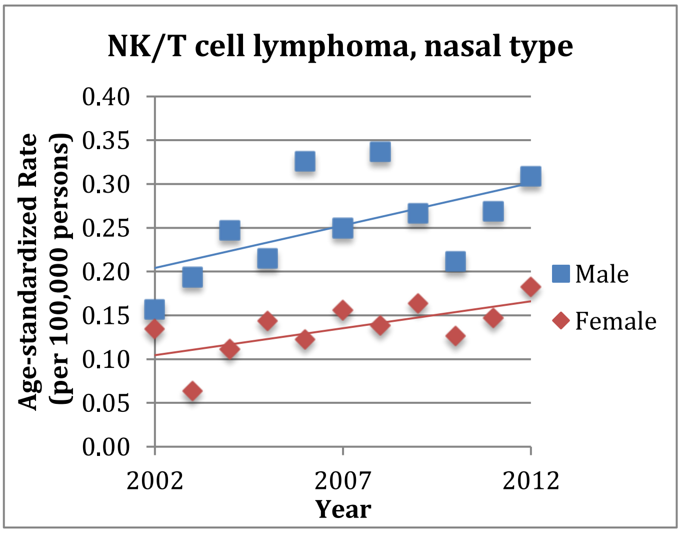

*Adult T-cell Leukemia/Lymphoma (ATLL) is not shown in this figure due to insufficient cases (< 3).

Figure S8. Age-standardized rate of HL and some NHL subtypes in American[1], Japanese[1] and Taiwanese male and female between the years 2002-2008

Hodgkin’s lymphoma: male

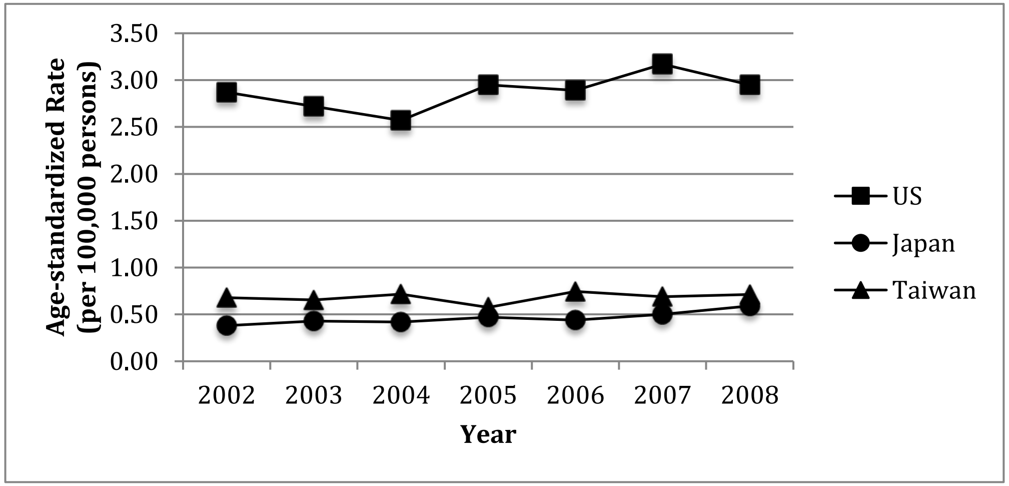

Hodgkin’s lymphoma: female

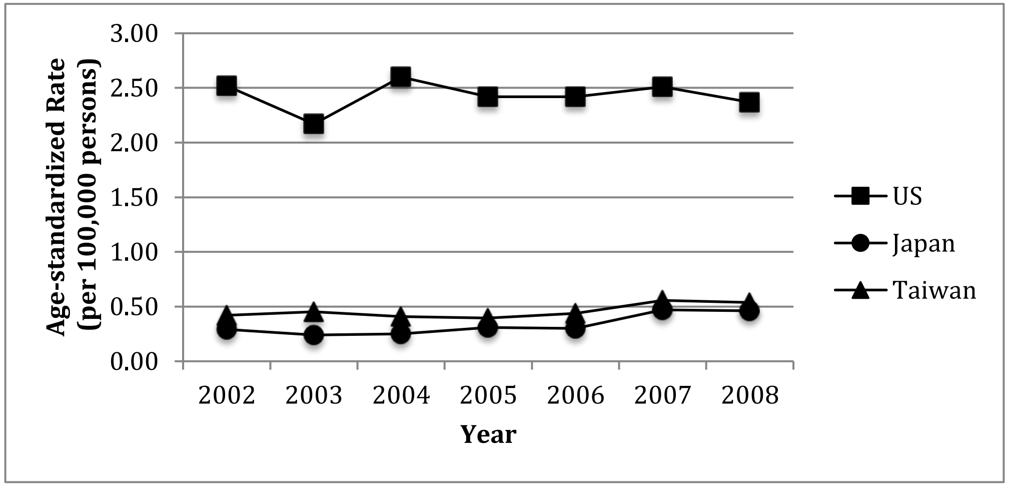

Mantle cell lymphoma: male

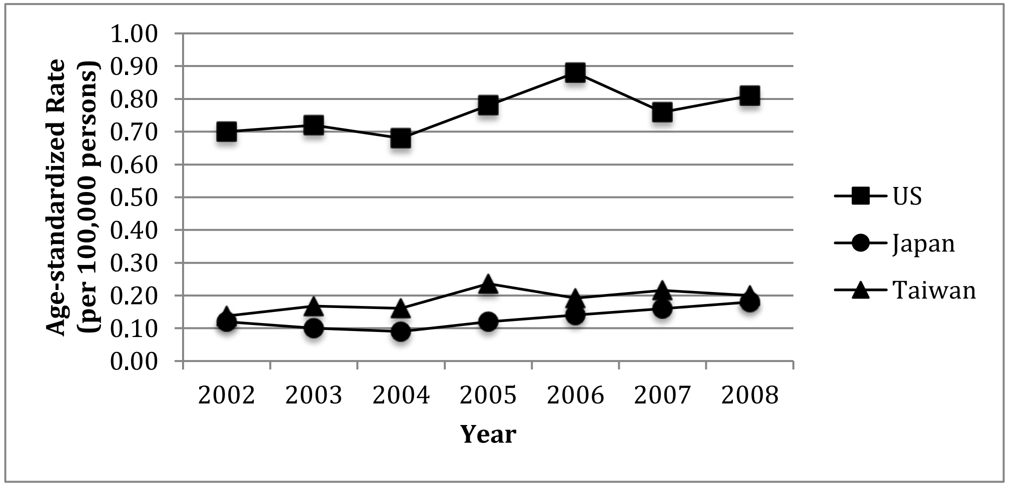

Mantle cell lymphoma: female

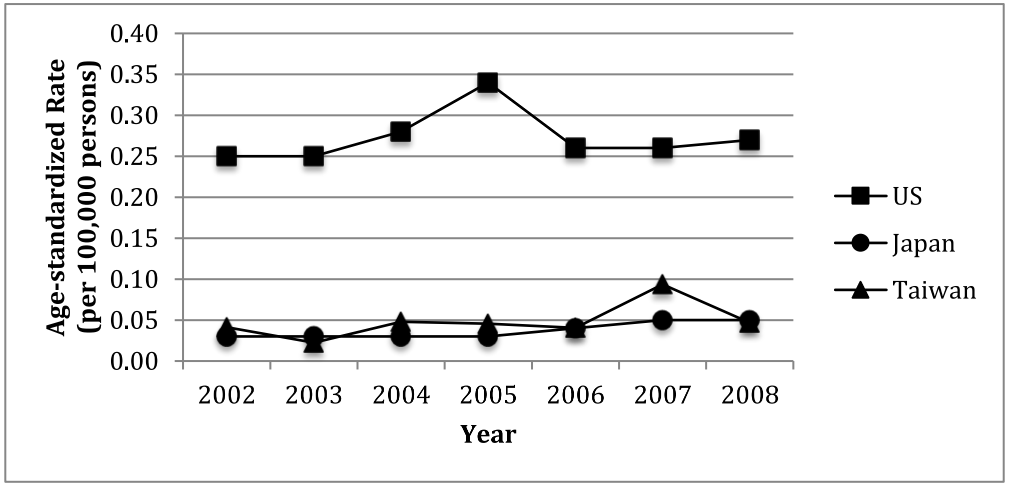

Figure S8. Age-standardized rate of HL and some NHL subtypes in American[1], Japanese[1] and Taiwanese male and female between the years 2002-2008 (*continued*)

Follicular lymphoma: male

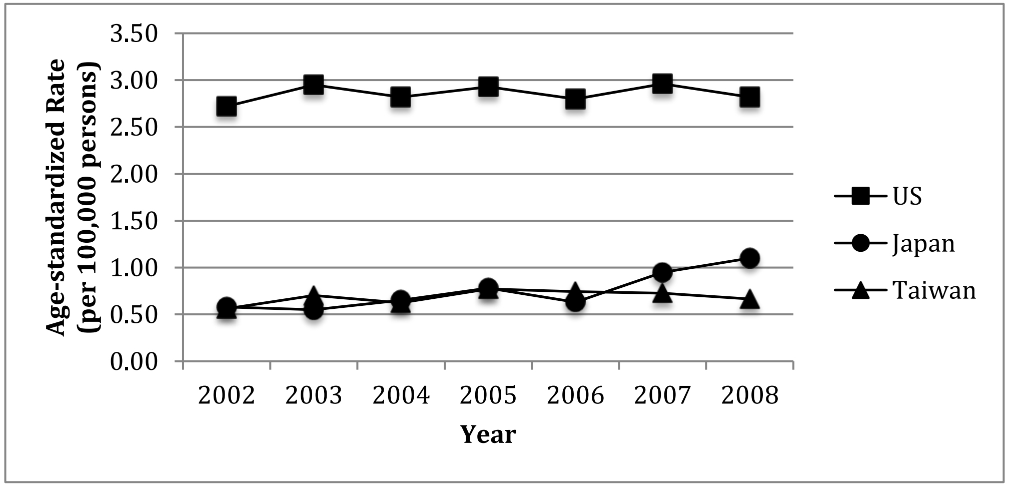

Follicular lymphoma: female

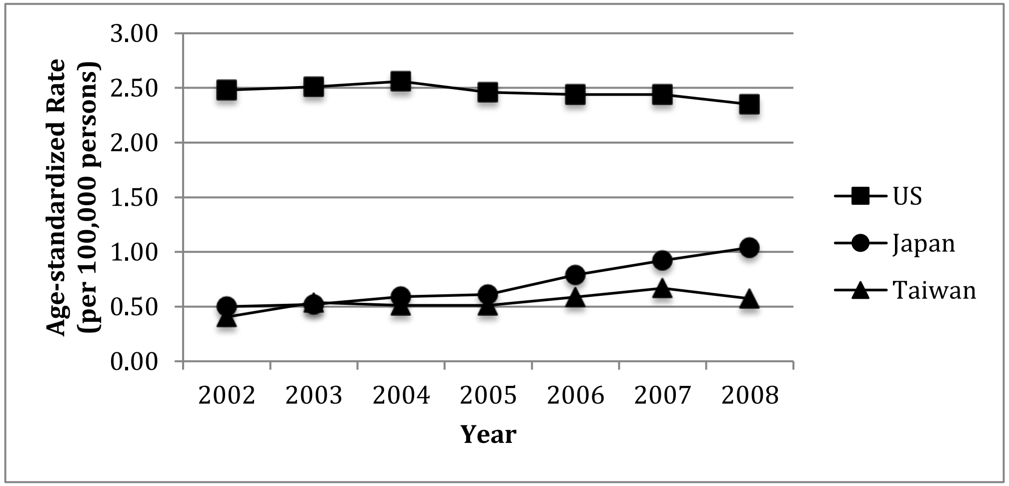

Chronic lymphocytic leukemia/ small lymphocytic lymphoma: male

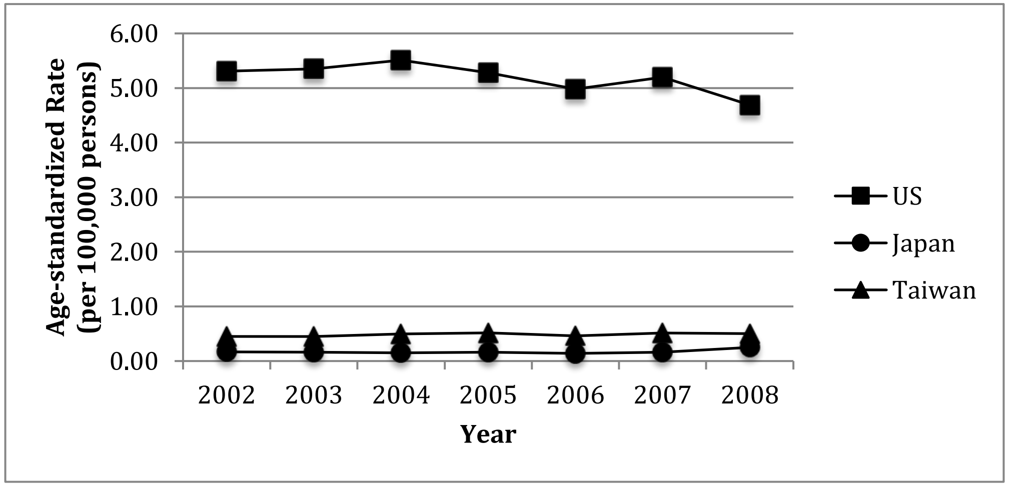

Chronic lymphocytic leukemia/ small lymphocytic lymphoma: female

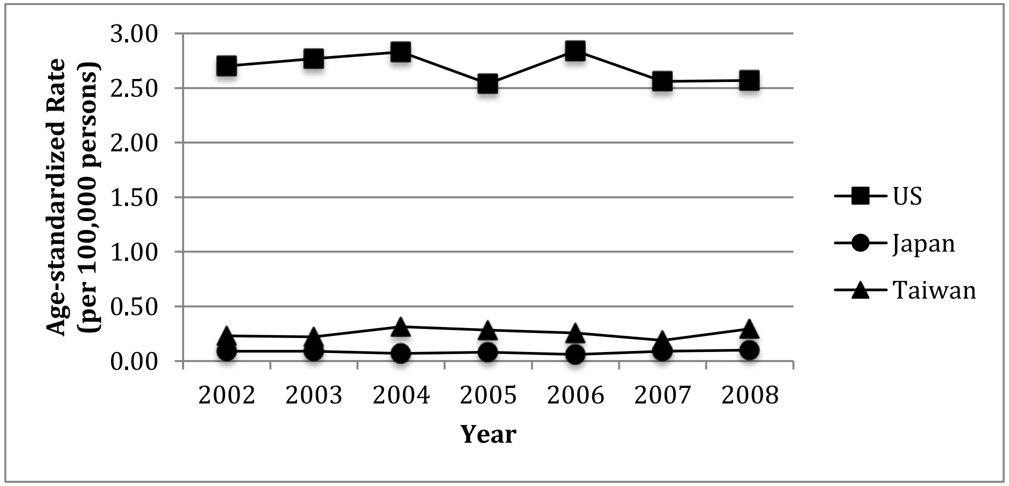

Figure S8. Age-standardized rate of HL and some NHL subtypes in American[1], Japanese[1] and Taiwanese male and female between the years 2002-2008 (*continued*)

Peripheral T-cell lymphoma-NOS: male

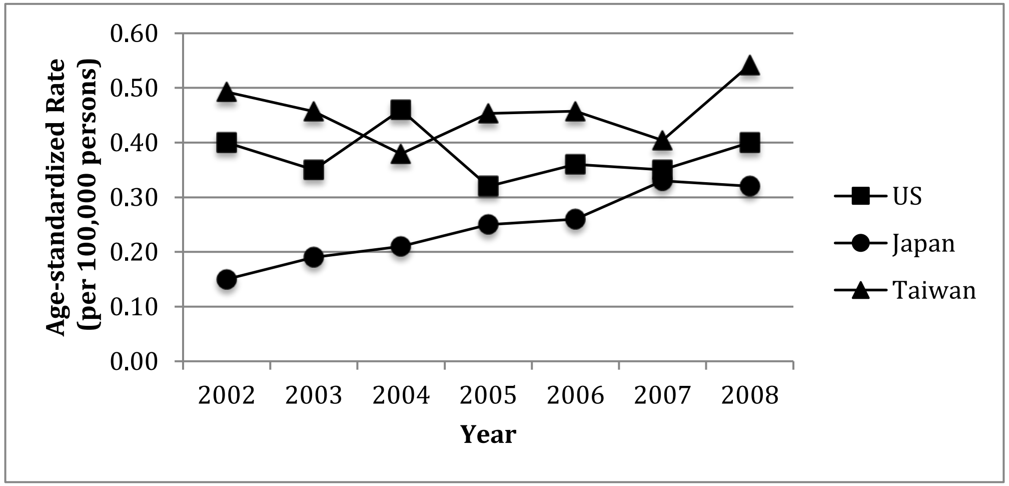

Peripheral T-cell lymphoma-NOS: female

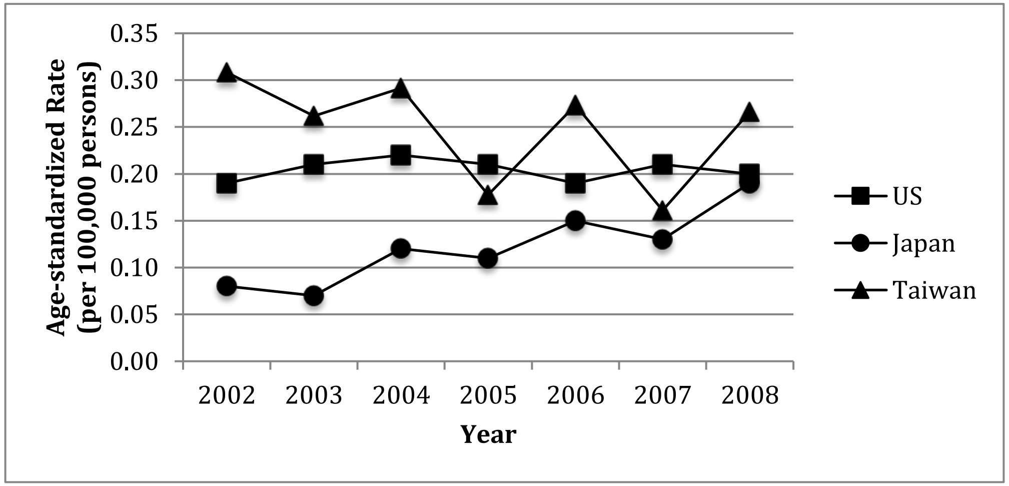

Mycosis fungoides: male

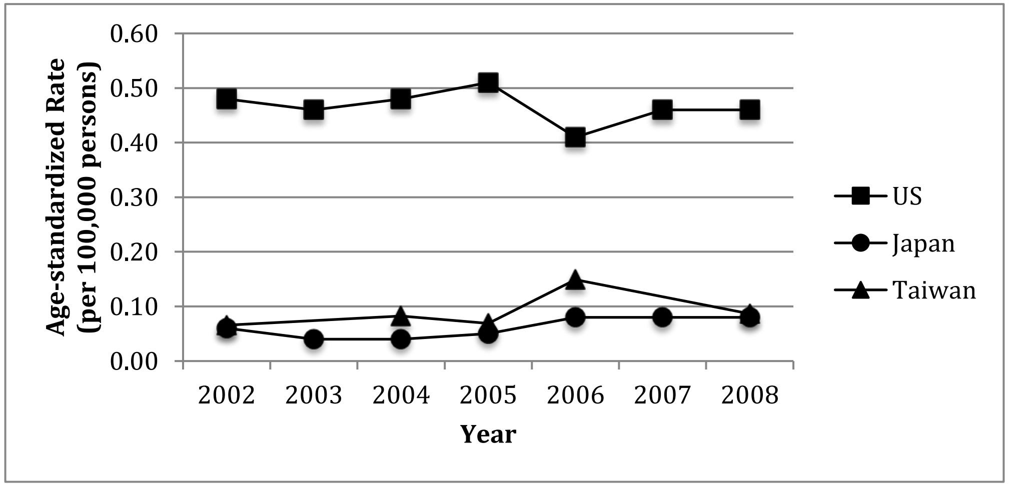

Mycosis fungoides: female

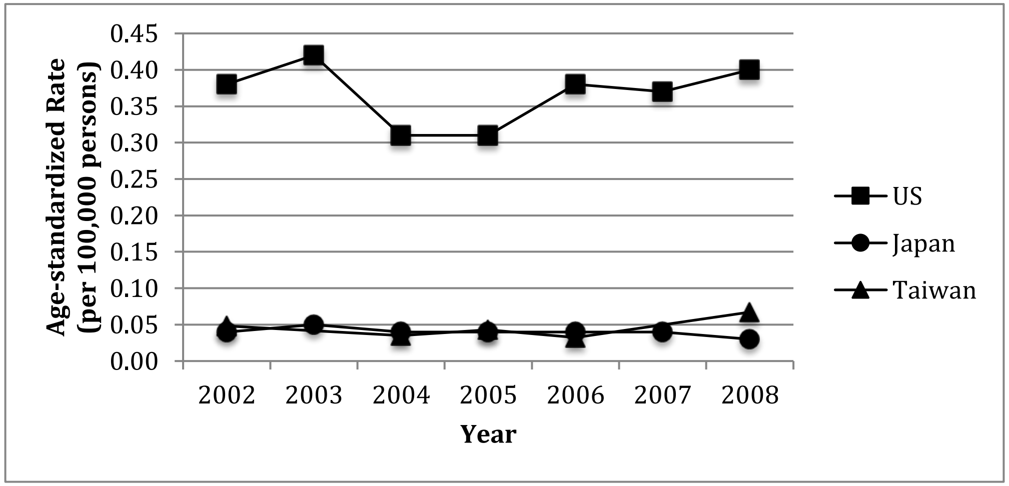

Figure S8. Age-standardized rate of HL and some NHL subtypes in American[1], Japanese[1] and Taiwanese male and female between the years 2002-2008 (*continued*)

Cutaneous T-cell lymphoma: male

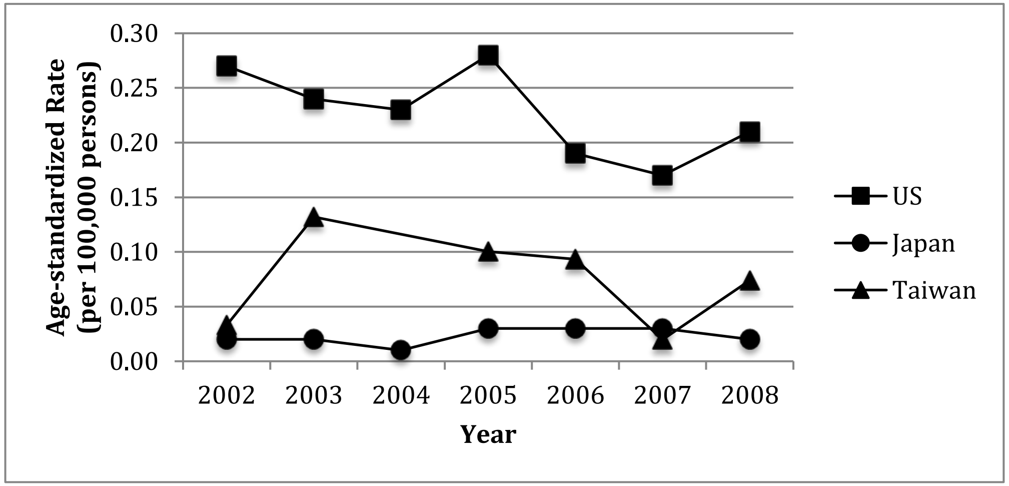

Cutaneous T-cell lymphoma: female

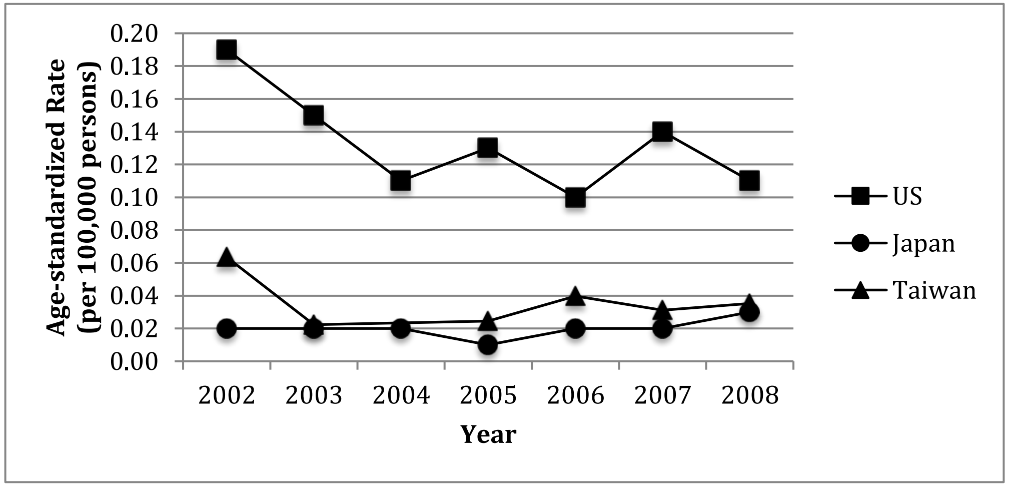

Anaplastic large T/null-cell lymphoma: male

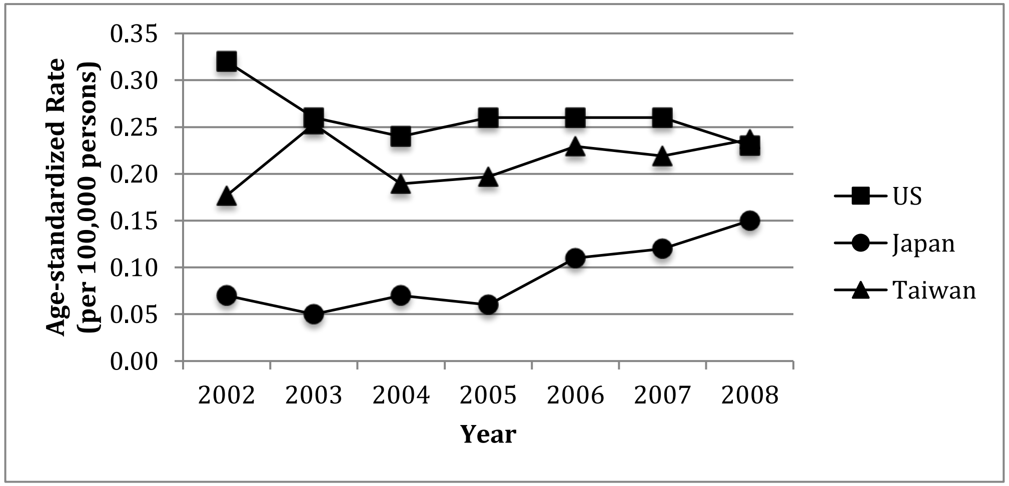

Anaplastic large T/null-cell lymphoma: female

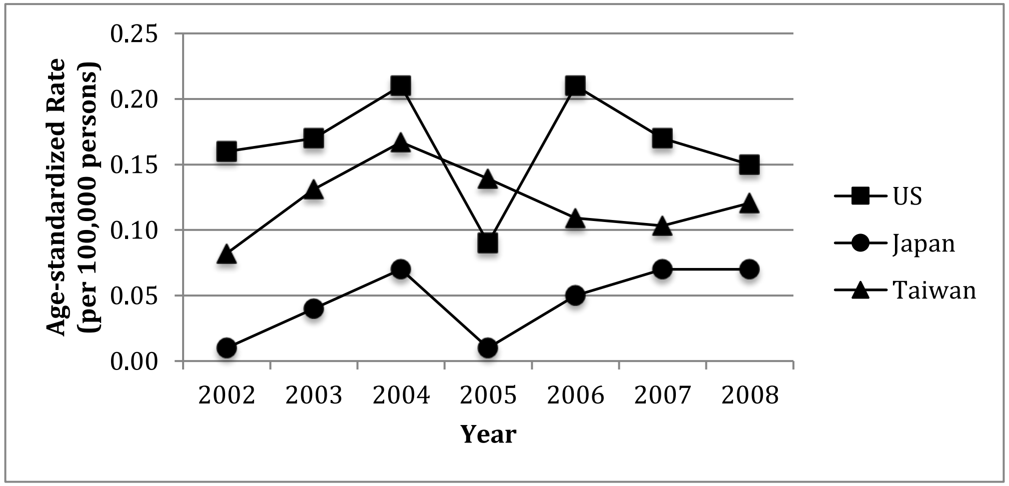

Figure S8. Age-standardized rate of HL and some NHL subtypes in American[1], Japanese[1] and Taiwanese male and female between the years 2002-2008 (*continued*)

Angioimmublastic T-cell lymphoma: male

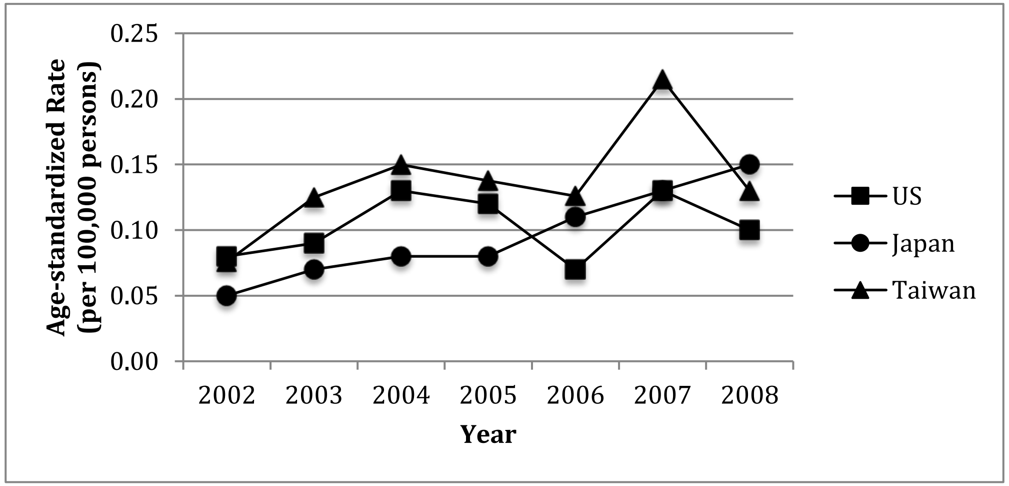

Angioimmublastic T-cell lymphoma: female

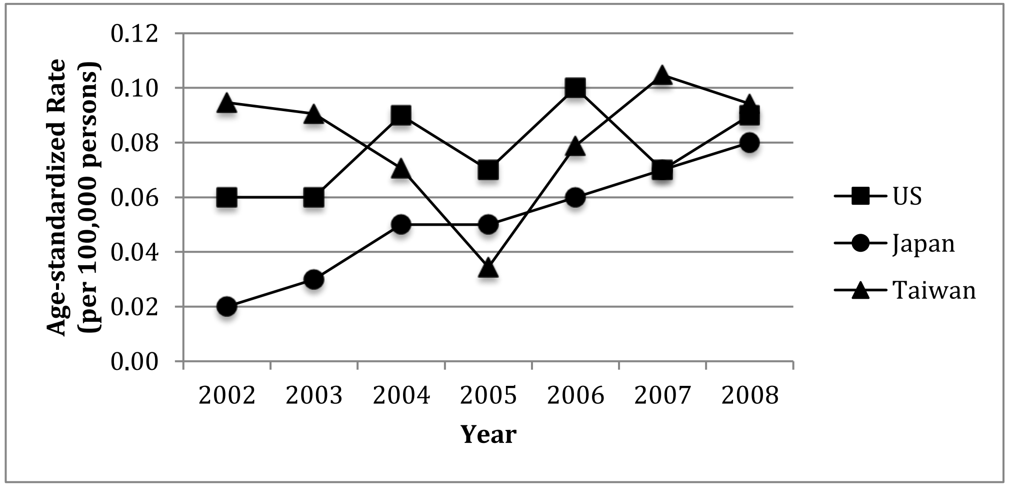

*Lack of Taiwanese data in certain lymphomas in some years due to insufficient cases defined as less than 3

Reference:

1. Chihara D, Ito H, Matsuda T, et al. Differences in incidence and trends of haematological malignancies in Japan and the United States. Br J Haematol 2013;164:536-45.
