# Supplementary material for: Subtype‐specific epidemiology of lymphoid malignancies in Taiwan compared to Japan and the United States, 2002‐2012
Source: Cancer Med. 2018 Oct 9;7(11):5820–31. doi: 10.1002/cam4.1762 (PMC6246924; doi:10.1002/cam4.1762)
Supplement: Supplementary file 2 [file CAM4-7-5820-s002.docx]

Table S1. Comparisons regarding ASRs of lymphomas among the US^1^, Japan^1^, and Taiwan between the years 2002 – 2008

| Disease | Number of incidence/  Age-standardized rate | 2002 | | 2003 | | 2004 | | 2005 | | 2006 | | 2007 | | 2008 | |
| --- | --- | --- | --- | --- | --- | --- | --- | --- | --- | --- | --- | --- | --- | --- | --- |
|  |  | male | female | male | female | male | female | male | female | male | female | male | female | male | female |
| Hodgkin’s lymphoma | Number of incidence - US | 429 | 372 | 409 | 333 | 402 | 403 | 465 | 371 | 447 | 387 | 501 | 388 | 466 | 371 |
|  | Age-standardized rate - US | 2.87 | 2.52 | 2.72 | 2.17 | 2.57 | 2.60 | 2.95 | 2.42 | 2.89 | 2.42 | 3.17 | 2.51 | 2.95 | 2.37 |
|  | Number of incidence - JP | 84 | 63 | 119 | 73 | 126 | 66 | 135 | 93 | 134 | 88 | 147 | 116 | 178 | 125 |
|  | Age-standardized rate - JP | 0.38 | 0.29 | 0.43 | 0.24 | 0.42 | 0.25 | 0.47 | 0.31 | 0.44 | 0.30 | 0.50 | 0.47 | 0.59 | 0.46 |
|  | Number of incidence - TW | 81 | 47 | 76 | 53 | 84 | 46 | 69 | 47 | 88 | 52 | 84 | 63 | 86 | 62 |
|  | Age-standardized rate - TW | 0.68 | 0.42 | 0.66 | 0.45 | 0.72 | 0.41 | 0.57 | 0.40 | 0.74 | 0.44 | 0.69 | 0.56 | 0.71 | 0.54 |
| Diffuse large B-cell lymphoma | Number of incidence - US | 989 | 829 | 1050 | 939 | 1084 | 945 | 1093 | 881 | 1050 | 863 | 1106 | 917 | 1088 | 910 |
|  | Age-standardized rate - US | 5.46 | 3.77 | 5.66 | 4.04 | 5.71 | 4.06 | 5.77 | 3.66 | 5.40 | 3.67 | 5.59 | 3.69 | 5.41 | 3.63 |
|  | Number of incidence - JP | 449 | 388 | 670 | 533 | 744 | 596 | 884 | 699 | 1070 | 817 | 1270 | 1035 | 1382 | 1109 |
|  | Age-standardized rate - JP | 1.50 | 1.14 | 1.73 | 1.21 | 1.83 | 1.33 | 2.11 | 1.36 | 2.46 | 1.64 | 2.92 | 2.07 | 3.06 | 1.99 |
|  | Number of incidence - TW | 364 | 305 | 395 | 322 | 407 | 326 | 443 | 338 | 450 | 364 | 470 | 393 | 492 | 373 |
|  | Age-standardized rate - TW | 2.95 | 2.58 | 3.14 | 2.66 | 3.11 | 2.58 | 3.37 | 2.59 | 3.26 | 2.71 | 3.35 | 2.80 | 3.44 | 2.57 |
| Burkitt lymphoma | Number of incidence - US | 60 | 29 | 58 | 26 | 77 | 19 | 82 | 19 | 70 | 36 | 91 | 41 | 91 | 32 |
|  | Age-standardized rate - US | 0.40 | 0.17 | 0.42 | 0.14 | 0.49 | 0.13 | 0.54 | 0.12 | 0.44 | 0.21 | 0.59 | 0.24 | 0.60 | 0.19 |
|  | Number of incidence - JP | 17 | 11 | 17 | 15 | 34 | 24 | 29 | 11 | 34 | 21 | 35 | 18 | 38 | 25 |
|  | Age-standardized rate - JP | 0.09 | 0.04 | 0.10 | 0.05 | 0.15 | 0.09 | 0.14 | 0.04 | 0.14 | 0.05 | 0.17 | 0.08 | 0.14 | 0.10 |
|  | Number of incidence - TW | 20 | 22 | 26 | 13 | 30 | 19 | 32 | 18 | 33 | 25 | 41 | 12 | 31 | 17 |
|  | Age-standardized rate - TW | 0.17 | 0.20 | 0.23 | 0.12 | 0.28 | 0.18 | 0.29 | 0.16 | 0.29 | 0.22 | 0.34 | 0.09 | 0.28 | 0.15 |

Table S1. Comparisons regarding ASRs of lymphomas among the US^1^, Japan^1^, and Taiwan between the years 2002 – 2008 (*continued*)

| Disease | Number of incidence/  Age-standardized rate | 2002 | | 2003 | | 2004 | | 2005 | | 2006 | | 2007 | | 2008 | |
| --- | --- | --- | --- | --- | --- | --- | --- | --- | --- | --- | --- | --- | --- | --- | --- |
|  |  | male | female | male | female | male | female | male | female | male | female | male | female | male | female |
| Mantle cell lymphoma | Number of incidence - US | 124 | 57 | 130 | 49 | 124 | 67 | 151 | 79 | 177 | 66 | 154 | 64 | 162 | 70 |
|  | Age-standardized rate - US | 0.70 | 0.25 | 0.72 | 0.25 | 0.68 | 0.28 | 0.78 | 0.34 | 0.88 | 0.26 | 0.76 | 0.26 | 0.81 | 0.27 |
|  | Number of incidence - JP | 35 | 9 | 39 | 18 | 38 | 13 | 53 | 16 | 64 | 20 | 72 | 23 | 90 | 26 |
|  | Age-standardized rate - JP | 0.12 | 0.03 | 0.10 | 0.03 | 0.09 | 0.03 | 0.12 | 0.03 | 0.14 | 0.04 | 0.16 | 0.05 | 0.18 | 0.05 |
|  | Number of incidence - TW | 18 | 5 | 19 | 3 | 22 | 6 | 30 | 6 | 26 | 6 | 29 | 13 | 29 | 7 |
|  | Age-standardized rate - TW | 0.14 | 0.04 | 0.17 | 0.02 | 0.16 | 0.05 | 0.24 | 0.05 | 0.19 | 0.04 | 0.22 | 0.09 | 0.20 | 0.05 |
| Follicular lymphoma | Number of incidence - US | 473 | 503 | 514 | 516 | 511 | 543 | 545 | 528 | 533 | 542 | 568 | 538 | 554 | 546 |
|  | Age-standardized rate - US | 2.72 | 2.48 | 2.95 | 2.51 | 2.82 | 2.56 | 2.93 | 2.46 | 2.80 | 2.44 | 2.96 | 2.44 | 2.82 | 2.35 |
|  | Number of incidence - JP | 149 | 138 | 177 | 175 | 218 | 207 | 266 | 217 | 230 | 274 | 331 | 340 | 389 | 401 |
|  | Age-standardized rate - JP | 0.58 | 0.50 | 0.55 | 0.52 | 0.65 | 0.59 | 0.78 | 0.61 | 0.63 | 0.79 | 0.95 | 0.92 | 1.10 | 1.04 |
|  | Number of incidence - TW | 68 | 50 | 90 | 68 | 80 | 64 | 104 | 70 | 102 | 81 | 100 | 93 | 94 | 83 |
|  | Age-standardized rate - TW | 0.56 | 0.41 | 0.70 | 0.54 | 0.62 | 0.51 | 0.77 | 0.51 | 0.74 | 0.59 | 0.72 | 0.67 | 0.66 | 0.57 |
| CLL/ SLL | Number of incidence - US | 953 | 664 | 992 | 685 | 1052 | 701 | 1027 | 651 | 1013 | 749 | 1061 | 664 | 995 | 698 |
|  | Age-standardized rate - US | 5.31 | 2.70 | 5.35 | 2.77 | 5.51 | 2.83 | 5.28 | 2.54 | 4.98 | 2.84 | 5.20 | 2.56 | 4.69 | 2.57 |
|  | Number of incidence - JP | 58 | 35 | 67 | 47 | 67 | 44 | 76 | 51 | 70 | 39 | 82 | 58 | 118 | 66 |
|  | Age-standardized rate - JP | 0.17 | 0.09 | 0.16 | 0.09 | 0.15 | 0.07 | 0.16 | 0.08 | 0.14 | 0.06 | 0.16 | 0.09 | 0.25 | 0.10 |
|  | Number of incidence - TW | 55 | 27 | 57 | 28 | 64 | 40 | 66 | 37 | 63 | 36 | 73 | 28 | 74 | 43 |
|  | Age-standardized rate - TW | 0.45 | 0.23 | 0.45 | 0.22 | 0.49 | 0.31 | 0.52 | 0.28 | 0.46 | 0.26 | 0.51 | 0.19 | 0.50 | 0.30 |

Table S1. Comparisons regarding ASRs of lymphomas among the US^1^, Japan^1^, and Taiwan between the years 2002 – 2008 (*continued*)

| Disease | Number of incidence/  Age-standardized rate | 2002 | | 2003 | | 2004 | | 2005 | | 2006 | | 2007 | | 2008 | |
| --- | --- | --- | --- | --- | --- | --- | --- | --- | --- | --- | --- | --- | --- | --- | --- |
|  |  | male | female | male | female | male | female | male | female | male | female | male | female | male | female |
| Marginal zone B-cell lymphoma | Number of incidence - US | 189 | 238 | 197 | 217 | 203 | 286 | 196 | 254 | 197 | 272 | 265 | 282 | 255 | 269 |
|  | Age-standardized rate - US | 1.01 | 1.12 | 1.09 | 1.02 | 1.09 | 1.23 | 1.05 | 1.08 | 1.02 | 1.19 | 1.36 | 1.14 | 1.26 | 1.06 |
|  | Number of incidence - JP | 52 | 62 | 70 | 106 | 99 | 106 | 103 | 133 | 118 | 144 | 185 | 193 | 208 | 242 |
|  | Age-standardized rate - JP | 0.22 | 0.20 | 0.20 | 0.25 | 0.26 | 0.27 | 0.29 | 0.33 | 0.25 | 0.33 | 0.25 | 0.44 | 0.25 | 0.52 |
|  | Number of incidence - TW | 67 | 63 | 58 | 53 | 52 | 79 | 79 | 60 | 70 | 83 | 101 | 95 | 90 | 108 |
|  | Age-standardized rate - TW | 0.55 | 0.53 | 0.46 | 0.43 | 0.40 | 0.63 | 0.61 | 0.45 | 0.53 | 0.61 | 0.71 | 0.66 | 0.61 | 0.75 |
| Peripheral T-cell lymphoma-NOS | Number of incidence - US | 62 | 45 | 61 | 45 | 84 | 47 | 60 | 43 | 68 | 42 | 70 | 52 | 78 | 46 |
|  | Age-standardized rate - US | 0.40 | 0.19 | 0.35 | 0.21 | 0.46 | 0.22 | 0.32 | 0.21 | 0.36 | 0.19 | 0.35 | 0.21 | 0.40 | 0.20 |
|  | Number of incidence - JP | 45 | 28 | 69 | 36 | 71 | 50 | 97 | 54 | 106 | 67 | 139 | 72 | 130 | 86 |
|  | Age-standardized rate - JP | 0.15 | 0.08 | 0.19 | 0.07 | 0.21 | 0.12 | 0.25 | 0.11 | 0.26 | 0.15 | 0.33 | 0.13 | 0.32 | 0.19 |
|  | Number of incidence - TW | 60 | 36 | 58 | 31 | 49 | 37 | 59 | 22 | 60 | 36 | 55 | 22 | 75 | 38 |
|  | Age-standardized rate - TW | 0.49 | 0.31 | 0.46 | 0.26 | 0.38 | 0.29 | 0.45 | 0.18 | 0.46 | 0.27 | 0.40 | 0.16 | 0.54 | 0.27 |
| Mycosis fungoides | Number of incidence - US | 84 | 66 | 80 | 77 | 85 | 56 | 91 | 61 | 78 | 70 | 84 | 74 | 86 | 82 |
|  | Age-standardized rate - US | 0.48 | 0.38 | 0.46 | 0.42 | 0.48 | 0.31 | 0.51 | 0.31 | 0.41 | 0.38 | 0.46 | 0.37 | 0.46 | 0.40 |
|  | Number of incidence - JP | 17 | 10 | 17 | 17 | 13 | 12 | 18 | 16 | 27 | 15 | 36 | 15 | 29 | 16 |
|  | Age-standardized rate - JP | 0.06 | 0.04 | 0.04 | 0.05 | 0.04 | 0.04 | 0.05 | 0.04 | 0.08 | 0.04 | 0.08 | 0.04 | 0.08 | 0.03 |
|  | Number of incidence - TW | 9 | 6 | * | * | 11 | 4 | 10 | 5 | 19 | 4 | * | * | 12 | 9 |
|  | Age-standardized rate - TW | 0.07 | 0.05 | * | * | 0.08 | 0.03 | 0.07 | 0.04 | 0.15 | 0.03 | * | * | 0.09 | 0.07 |

Table S1. Comparisons regarding ASRs of lymphomas among the US^1^, Japan^1^, and Taiwan between the years 2002 – 2008 (*continued*)

| Disease | Number of incidence/  Age-standardized rate | 2002 | | 2003 | | 2004 | | 2005 | | 2006 | | 2007 | | 2008 | |
| --- | --- | --- | --- | --- | --- | --- | --- | --- | --- | --- | --- | --- | --- | --- | --- |
|  |  | male | female | male | female | male | female | male | female | male | female | male | female | male | female |
| Cutaneous T-cell lymphoma | Number of incidence - US | 48 | 40 | 43 | 32 | 38 | 23 | 52 | 29 | 35 | 23 | 33 | 31 | 39 | 24 |
|  | Age-standardized rate - US | 0.27 | 0.19 | 0.24 | 0.15 | 0.23 | 0.11 | 0.28 | 0.13 | 0.19 | 0.10 | 0.17 | 0.14 | 0.21 | 0.11 |
|  | Number of incidence - JP | 5 | 7 | 5 | 8 | 5 | 8 | 11 | 4 | 11 | 8 | 13 | 6 | 9 | 13 |
|  | Age-standardized rate - JP | 0.02 | 0.02 | 0.02 | 0.02 | 0.01 | 0.02 | 0.03 | 0.01 | 0.03 | 0.02 | 0.03 | 0.02 | 0.02 | 0.03 |
|  | Number of incidence - TW | 4 | 8 | 15 | 3 | * | * | 14 | 3 | 12 | 5 | 3 | 4 | 9 | 5 |
|  | Age-standardized rate - TW | 0.03 | 0.06 | 0.13 | 0.02 | * | * | 0.10 | 0.02 | 0.09 | 0.04 | 0.02 | 0.03 | 0.07 | 0.04 |
| Anaplastic large T/null-cell lymphoma | Number of incidence - US | 50 | 23 | 40 | 31 | 37 | 35 | 43 | 18 | 40 | 35 | 45 | 33 | 38 | 30 |
|  | Age-standardized rate - US | 0.32 | 0.16 | 0.26 | 0.17 | 0.24 | 0.21 | 0.26 | 0.09 | 0.26 | 0.21 | 0.26 | 0.17 | 0.23 | 0.15 |
|  | Number of incidence - JP | 17 | 4 | 16 | 10 | 23 | 17 | 25 | 3 | 30 | 16 | 39 | 20 | 42 | 23 |
|  | Age-standardized rate - JP | 0.07 | 0.01 | 0.05 | 0.04 | 0.07 | 0.07 | 0.06 | 0.01 | 0.11 | 0.05 | 0.12 | 0.07 | 0.15 | 0.07 |
|  | Number of incidence - TW | 21 | 9 | 31 | 14 | 22 | 20 | 23 | 16 | 28 | 13 | 27 | 12 | 30 | 16 |
|  | Age-standardized rate - TW | 0.18 | 0.08 | 0.25 | 0.13 | 0.19 | 0.17 | 0.20 | 0.14 | 0.23 | 0.11 | 0.22 | 0.10 | 0.24 | 0.12 |
| Angio- immuno- blastic T-cell lymphoma | Number of incidence - US | 14 | 10 | 17 | 14 | 23 | 21 | 20 | 16 | 12 | 22 | 25 | 16 | 19 | 22 |
|  | Age-standardized rate - US | 0.08 | 0.06 | 0.09 | 0.06 | 0.13 | 0.09 | 0.12 | 0.07 | 0.07 | 0.10 | 0.13 | 0.07 | 0.10 | 0.09 |
|  | Number of incidence - JP | 14 | 7 | 30 | 16 | 37 | 29 | 37 | 31 | 51 | 30 | 60 | 44 | 68 | 50 |
|  | Age-standardized rate - JP | 0.05 | 0.02 | 0.07 | 0.03 | 0.08 | 0.05 | 0.08 | 0.05 | 0.11 | 0.06 | 0.13 | 0.07 | 0.15 | 0.08 |
|  | Number of incidence - TW | 9 | 11 | 16 | 11 | 19 | 9 | 18 | 5 | 16 | 11 | 28 | 15 | 19 | 14 |
|  | Age-standardized rate - TW | 0.08 | 0.09 | 0.12 | 0.09 | 0.15 | 0.07 | 0.14 | 0.03 | 0.13 | 0.08 | 0.21 | 0.10 | 0.13 | 0.09 |

Table S1. Comparisons regarding ASRs of lymphomas among the US^1^, Japan^1^, and Taiwan between the years 2002 – 2008 (*continued*)

| Disease | Number of incidence/  Age-standardized rate | 2002 | | 2003 | | 2004 | | 2005 | | 2006 | | 2007 | | 2008 | |
| --- | --- | --- | --- | --- | --- | --- | --- | --- | --- | --- | --- | --- | --- | --- | --- |
|  |  | male | female | male | female | male | female | male | female | male | female | male | female | male | female |
| NK/T-cell lymphoma, nasal type | Number of incidence - US | 12 | 4 | 8 | 5 | 5 | 3 | 14 | 5 | 9 | 6 | 11 | 8 | 17 | 2 |
|  | Age-standardized rate - US | 0.07 | 0.02 | 0.04 | 0.02 | 0.03 | 0.02 | 0.09 | 0.03 | 0.05 | 0.03 | 0.07 | 0.04 | 0.09 | 0.01 |
|  | Number of incidence - JP | 9 | 7 | 16 | 8 | 19 | 16 | 29 | 19 | 33 | 16 | 33 | 26 | 39 | 21 |
|  | Age-standardized rate - JP | 0.04 | 0.03 | 0.04 | 0.04 | 0.05 | 0.04 | 0.08 | 0.05 | 0.10 | 0.05 | 0.11 | 0.08 | 0.11 | 0.06 |
|  | Number of incidence - TW | 20 | 17 | 24 | 8 | 32 | 14 | 29 | 19 | 43 | 17 | 34 | 21 | 46 | 20 |
|  | Age-standardized rate - TW | 0.16 | 0.13 | 0.19 | 0.06 | 0.25 | 0.11 | 0.22 | 0.14 | 0.33 | 0.12 | 0.25 | 0.16 | 0.34 | 0.14 |

-Adult T-cell Leukemia/Lymphoma (ATLL) is not shown in this table due to insufficient cases (< 3).

-The symbol “*” in the table indicates insufficient cases defined as less than 3.

Reference:

1. Chihara D, Ito H, Matsuda T, et al. Differences in incidence and trends of haematological malignancies in Japan and the United States. Br J Haematol 2013;164:536-45.
